# Two routes to a target: Visual priming for direct and indirect attentional sets

**DOI:** 10.3758/s13421-025-01826-6

**Published:** 2025-12-15

**Authors:** Alexander Pastukhov, Malin Styrnal, Claus-Christian Carbon, Árni Kristjánsson

**Affiliations:** 1 Department of General Psychology and Methodology, University of Bamberg, Bamberg, Bavaria, Germany; 2 Research Group EPÆG (Ergonomics, Psychological Æsthetics, Gestalt), Bamberg, Bavaria, Germany; 3 Department of Medicine, University of Giessen, Giessen, Germany; 4 Icelandic Vision Lab, University of Iceland, Nýi Garður 207, 101 Reykjavík, Iceland

**Keywords:** Repetition priming, Attentional sets, Conjunction search, Visual search

## Abstract

To find an item of interest among candidate objects we are directed by attentional sets that reflect our expectations and intentions and may also vary by whether items should be attended or ignored. We investigated how different attentional sets influence target search and the effect of prior experience on these attentional sets. Our participants had to identify a target object given a set of objects that either contained the target itself (*direct* attentional set) or contained only cues that defined the target by exclusion (*indirect* attentional set). We found that response times were significantly slower for indirect attentional sets and when sets were mixed within blocks. To analyze the impact of attentional sets on priming, we fitted behavioral time series using multiple dynamic ideal observer models based on a first-order memory mechanism with three consecutive stages: set identification (direct vs. indirect), target identification (based on set cues), and response. The different models involved different assumptions about each stage, and we compared them via information criterion to identify mechanisms that consistently lead to good expected out-of-sample performance. We found strong repetition priming when both set and target were repeated. For direct attentional set, repetition priming was consistent with a first-order memory mechanism that tracks objects and colors likely connected to feature-specific neural mechanisms and frontoparietal attention network. In contrast, the processing of indirect attentional sets relies on qualitatively different mechanisms and search strategies than conventional visual search, likely related to neural networks involved in task switching and generation of attentional set.

**Supplementary Information:**

The online version contains supplementary material available at 10.3758/s13421-025-01826-6.

## Introduction

We use attention to select the items in the environment that match our goals for further processing. *Attentional set* is a concept introduced by Mowrer (1938) that is often used to describe the guidance of our attention or setting up of expectations (Gibson, 1941; Neisser et al., 1963; for reviews, see Driver, 2001; Kristjánsson & Egeth, 2020). Our attentional set can guide us towards or away from the features or objects of interest at a given moment. Let us say that you are looking for a mug in the communal lab kitchen. The attentional set may specify the cup – your red Liverpool FC mug – or a mug that has a cylindrical shape, the color red, and perhaps the YNWA letter combination, so any mug that satisfies these criteria will do. Conversely, your colleague may want to avoid anything connected with Manchester United and, thus, avoids certain combinations of letters and colors at all costs. Or you might be looking for the most unique mug (an oddball mug) or the most typical mug (so you can blend in pretending that you are a football fan too). Depending on the context and specific information within each attentional set, these people with different goals may end up choosing the same mug but their choice processes will be very different.

We are very flexible in setting up and adjusting attentional sets based on the current context and task demands. In the simplest case, an attentional set can define expectations about the target. For example, Sekuler and Ball (1977) used a task where observers had to decide which of two temporal intervals contained moving dots (the other interval was blank). Sekuler and Ball adjusted the contrast of the dots against their background until performance on the task reached about 75% correct when the movement of the dots was in the same direction on every trial. For those same luminance conditions, performance was near chance (55% correct) when the direction of the moving dots was random from trial to trial. Thus, a more specific attentional set – tuned not just to motion but also to a particular drift direction – produced a dramatic increase in performance.

Attentional sets can also include a particular location, unexpected events, or certain feature values (Egeth & Yantis, 1997). Bacon and Egeth (1994) showed that observers can have a set for detecting unexpected singletons of any type (a set for singleton detection) or can have their attentional set tuned more narrowly so irrelevant singletons will not capture attention. When observers simply needed to identify an object with an odd shape (a circle target among diamond non-targets), their search performance was disrupted by the presence of a uniquely colored non-target diamond, a so-called attentional capture effect (Theeuwes, 1992). But when they adjusted their attentional set to exclude an irrelevant distractor, the effects of this “singleton detection mode” were eliminated. This shows the variable nature of attentional sets (see further evidence in Lamy & Egeth, 2003), and is related to the well-known distinction made by Shiffrin and Schneider (1977) between automatic and controlled search processes where attention can work as a filter so that certain aspects (e.g., features or locations) can be ignored and other aspects can be highlighted.

Attentional sets are determined not just by the instructions and the goals but also by our prior experience. For example, a lot of studies in the visual attention literature show how our attention is drawn to the items that we have just attended to, i.e., the target object is automatically included into the attentional set for the following trial. Accordingly, target identification is faster and more accurate if the search targets have the same color as during previous selections or if they appear in the same locations as during previous selections (for review, see Kristjánsson & Ásgeirsson, 2019). In fact, this adds to a large amount of literature showing how important temporal context is for perception – which reflects the way the visual system uses the past to predict the present (Chetverikov et al., 2016; Chetverikov & Kristjansson, 2015; Kristjánsson, 2023; Lin & Qian, 2024; Pascucci et al., 2023; Rao & Ballard, 1999; Seriès & Seitz, 2013; Tanrıkulu et al., 2021). A notable aspect of attentional priming patterns is the gradual build-up of priming and the time constant of this build-up (Brascamp et al., 2011; Martini, 2010).

However, there has been far less research into what might be called the priming of attentional sets themselves. How easily can such sets be generated and varied and how are they maintained over time? In Leber and Egeth (2006), observers could, during a training phase, use one of two different attentional sets (they could not use both) to identify targets in a stream of briefly presented items. In a subsequent test phase, observers had a strong tendency to persist in using the set they had used during the training phase. Fuggetta et al. (2009) then demonstrated priming of the size of the attentional window, as performance was better when the size of the selection area remained constant across trials than when it was variable. Relatedly, it is well documented that expectations about upcoming stimuli strongly influence performance: if targets in a visual search task suddenly become part of the distractor set, role-reversal effects occur where the search becomes slower and less accurate (Chetverikov & Kristjansson, 2015; Kristjánsson & Driver, 2008; Lamy et al., 2008).

When discussing attentional guidance, it is just as important to consider how visual cognition treats items that should not be attended or ignored. Therefore, while we may have a template for a target that we look for, we also can have templates for rejection (Arita et al., 2012; Carlisle, 2023; Carlisle & Kristjánsson, 2018; Cunningham & Egeth, 2016)*.* One example, familiar to many, is the classic wine bottle problem, where we have a bottle of wine with a cork firmly in place, but no corkscrew. We frantically hunt for something that can help us to open the bottle, without knowing exactly what that might be, finding a lot of things that will not work since they are not of the correct shape or functionality and therefore meet the exclusion criteria.

The aim of this study was to (1) test whether priming operates at the level of attentional sets themselves, beyond priming of sensory features such as shapes and colors, and (2) examine the mechanisms of negative attentional sets, specifically whether they rely on priming processes similar to those known for direct attentional sets. To this end, we examined performance across sequences of trials depending on stability of target shape, color, or attentional set, and quantified these effects using a family of ideal observer models. Our paradigm is particularly well-suited to disentangling the dynamics of direct and indirect attentional sets and may open pathways for future neural-level investigations. This work provides insights into how attentional sets are established, primed, and updated from trial to trial, including situations where observers know what the target is *not*.

## Methods

### Participants

All participants except one (the second author) were naïve to the purpose of the studies and all had normal or corrected-to-normal vision. All participants gave written consent. All procedures were in accordance with the national ethical standards on human experimentation and with the Declaration of Helsinki of 1975, as revised in 2008. Participants in the *easy* difficulty condition (three objects in the game set) could receive course credits within the framework of a mandatory module of research participation in accordance with the guidelines of the University of Bamberg. Most participants in the *medium* (five objects in the game set) and *hard* (nine objects in the game set) difficulty conditions were members of the University of Iceland community.

There were ten participants in the *easy* difficulty condition (eight female participants, aged 19–57 years, mean age 29 years, and two male participants, aged 26 and 57 years). Ten participants (five female participants, age 25–57 years, mean age 35 years, and five male participants, age 15–57 years, mean age 28 years) participated in the *medium* difficulty condition and another ten (six female participants, age 25–34 years, mean age 29 years, and four male participants, age 25–38 years, mean age 29 years) participated in the *hard* difficulty condition.

The sample size (N = 10 per condition) was chosen based on both prior repetition priming research and statistical power considerations. Although the number of participants is modest, the study employed a within-subject design with several hundred trials per participant, yielding high measurement precision and sensitivity to detect effects. Prior studies of visual repetition priming using similar paradigms typically report large effect sizes (Cohen’s *d* > 0.8; Chetverikov et al., 2016; Chetverikov & Kristjansson, 2015). A power analysis for a within-subject *t*-test with *d* = 0.8 and α =.05 indicates that ten participants provide approximately 80% power, suggesting that our sample size is sufficient to detect priming effects of magnitudes commonly observed in this domain.

We suspect that one participant (*rtgtirei*) in the *easy* difficulty condition misunderstood the instruction for blocks that contained both direct and indirect attentional sets and assumed that they corresponded to indirect search only (see below for details on the procedure), as their performance in this condition was below chance at 0% (i.e., responses were not random). However, as the participant performed normally in the three other conditions, we opted to keep the data for this participant in all but that specific condition.

### Apparatus

The experiment was programmed using oTree (Chen et al., 2016). Data analysis was performed using R (R Core Team, 2023) and the tidyverse set of packages (Wickham et al., 2019). Statistical models were coded and fitted with the Stan probabilistic programming language (Carpenter et al., 2017). Models were compared via a leave-one-out information criterion using the loo library (Vehtari et al., 2017). We used Microsoft Office icons as pictograms. The stimuli were presented on a 61-cm diagonal Eizo CG245W screen, resolution 1,920 × 1,200 and with a refresh rate of 60 Hz. A viewing distance of 50 cm was ensured by a chin-and-forehead rest that stabilized the viewing position and angle. A single pixel subtended 0.03 degrees of visual angle (dva). Participants responded on the keyboard.

### Experimental procedure

Our experimental design was based on a board game *Geistesblitz* (author Jacques Zeimet, illustrations by Gabriela Silveira, © 2010 Zoch Verlag, https://www.zoch-verlag.com/zoch_de/kategorien/familienspiele/geistesblitz-601129800-de.html). The main distinction is that our experiment was restricted to only two kinds of attentional sets determining target identity, whereas in the game, depending on the desired difficulty, a target can be identified based on up to five different kinds of attentional sets.

**Fig. 1:**
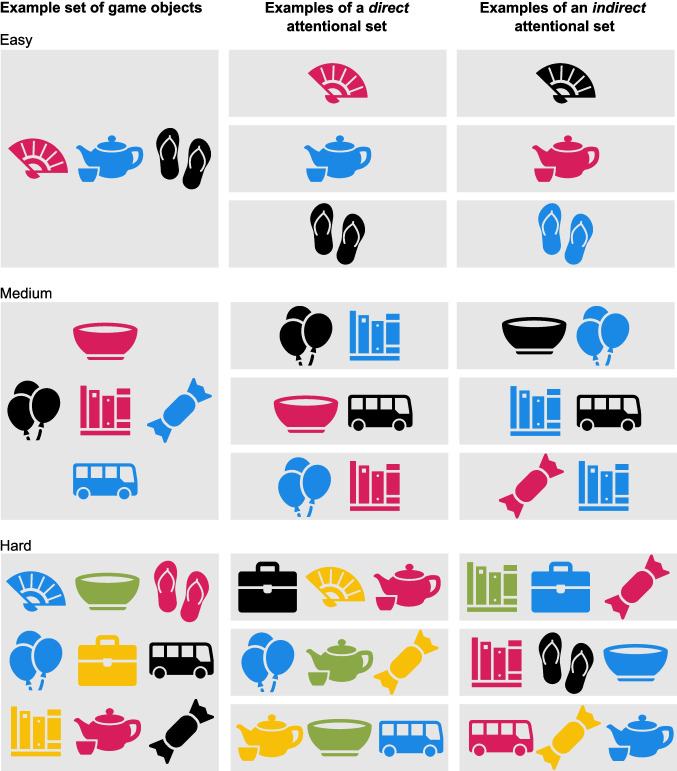
Example game objects (**left column**) and examples of direct (**middle column**) and indirect (**right column**) attentional sets as indicated by cue cards. For the *direct attentional set*, the target object has the correct color, i.e., there is one object that fully matches an object from the game objects set in the left column. For example, it is the red fan for the first example direct set for *easy* difficulty. For the *indirect attentional set*, the target object is not in the trial set and does not have any of the colors that are present in the set. For the first indirect attention set example for *easy* difficulty, the target object is *not* a fan and it is *not* a black object, which leaves the only remaining game set object: blue tea pot. The solutions for the remaining examples are given in Table 1

On each trial, participants had to identify a target object based on a smaller trial-specific set of objects (respectively, one, two, or three) that was either *direct* or *indirect*, see *Direct attentional set* and *Indirect attention set* panels in Fig. 1. The identity of a target object, accompanying distractors, and attention set were fully randomized. On each trial, only one object from the gaming set matched both the shape and color of the object. In the case of the *direct* attentional set, all but one of the objects were depicted in a mismatching color. For example, the first example of a direct attentional set for the *hard* difficulty condition in Fig. 1 contains a black briefcase (color of the briefcase in the game objects set is yellow), a yellow fan (color of the fan in the game objects set is blue), and a red tea pot (also red in the game objects set). The target object is therefore the red tea pot.

For the *indirect* attentional set, all objects within the trial attentional set were depicted in mismatching colors. For example, in the first example of the indirect attentional set for the *medium* difficulty in Fig. 1 the bowl is black (red in the game objects set), and balloons are blue (black in the game objects set). Thus in this case *all* objects are depicted in mismatching colors, so the target object needs to be identified by exclusion: it is not an object that is present in the trial set, which excludes the bowl and the balloons, and not of the color present in the set, which excludes all black (balloons) and blue (the bus and the candy) objects, leaving a single remaining object – the books. The reader can use these rules to identify target objects in the example sets in Fig. 1 with answers provided in Table 1 at the end of this section.

**Table 1:** Solutions to examples in Fig. 1

Difficulty	Direct attentional set	Indirect attention set
Easy	Fan, tea pot, flip flops	Tea pot, flip flops, fan
Medium	Balloons, bowl, books	Books, bowl, balloons
Hard	Tea pot, balloons, bowl	Bus, briefcase, bowl

For all three difficulty conditions, we used an ABBA block design with eight blocks of 80 trials each. The first and last block consisted only of trials with direct attentional sets. The second and seventh blocks included only indirect attentional sets. Blocks three to six consisted of trials with direct and indirect attentional sets in random order (i.e., (1) direct only, (2) indirect only, (3) mixed, (4) mixed, (5) mixed, (6) mixed, (7) indirect only, (8) direct only). Participants were given instructions on whether during the next block the attentional set would be only direct, only indirect, or mixed. As both target and attentional set were randomly selected on each trial, the number of repetitions on successive trials of the target object, attentional set, or both varied across participants and conditions (see Table 2).

**Table 2:** Number of average repetitions of the target object, attentional set, or both for each difficulty condition (mean ± standard deviation)

Difficulty	Repetitions of		
	Target object	Attentional set	Target and attentional set
Easy	288 ± 10	477 ± 11	218 ± 11
Medium	291 ± 16	474 ± 9	216 ± 17
Hard	251 ± 10	478 ± 9	191 ± 12

In the experiment, participants were presented with a set of game objects: three for the *easy* difficulty, five for the *medium* difficulty, and nine for the *hard* difficulty conditions (see *Game objects* panel in Fig. 1). The same objects and colors were used within each experimental session. The color and position of objects were randomized between participants but were constant for each participant throughout the entire experiment. For example, for the *hard* difficulty example in Fig. 1, once the bus object was assigned a black color and mid-right position for a particular participant, it would be used for the entire duration of experiment. A different participant would get a different combination, for example, a red bus in the top-left position, but this would be consistent for that participant across the entire experimental session. The constant position was required to simplify response mapping between keys and objects. For *easy* difficulty (three game objects), participants used the left, down, or right arrow keys; for *medium* difficulty (five game objects) they used the *W*, *A*, *S*, *D,* or *X* keys; for *hard* difficulty (nine game objects), participants used the numeric pad keys from 1 to 9.

**Fig. 2:**
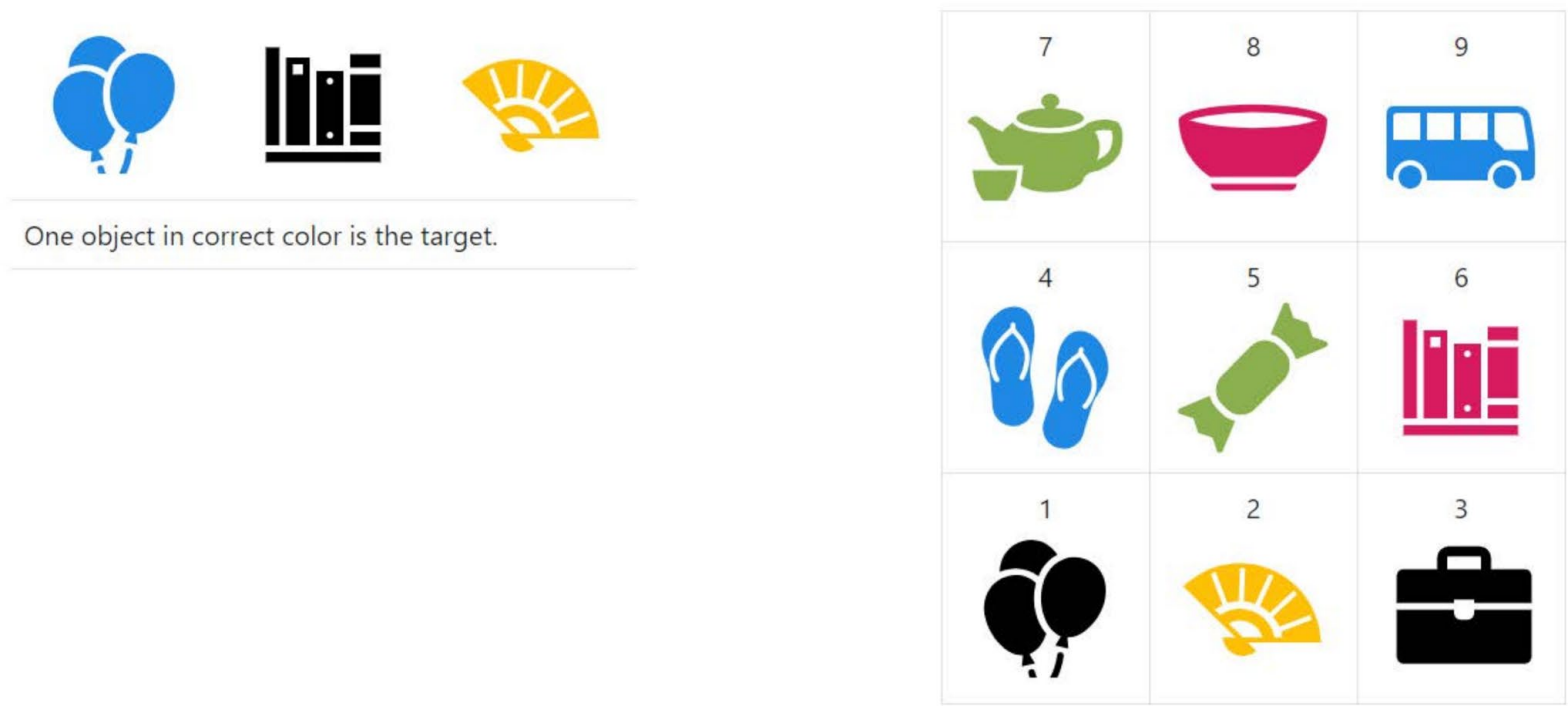
Example display for the *hard* difficulty condition, direct attentional set block. **Left:** attentional set. **Right:** game objects set

Each block started with instructions and information about attentional sets (only direct, only indirect, and mixed). Participants were informed that they could use the instruction stage for a break but would not be able to pause during the block. The game objects set was presented continuously throughout the entire block. Presentation of the trial set (objects that identified the target on this trial) was delayed by a random amount of time (500–600 ms) and the stimuli remained on the screen until response. The response times were measured relative to the stimulus and trial set onset. Each stimulus measured 2.9 × 2.9 dva. The game objects were presented on the right and the attentional set on the left side of the screen (see Fig. 2). Participants received feedback on their performance after each trial. If they picked the correct object, the square around the object turned green. If they picked the wrong object, the square turned red, whereas the square around the correct one turned black.

### Statistical analysis

Posterior distributions for all models were sampled using Stan probabilistic programming language (Carpenter et al., 2017). We characterized uncertainty using 97% credible (for posterior distributions) and confidence (for behavioral data) intervals. Given that the exact value used for computing such intervals is arbitrary, we picked 97 because it is a prime number.

We summarized posterior distributions of parameters and posterior predictions using a 97% credible interval. A credible interval, also known as a compatibility interval, is a range that contains a predefined proportion of the probability mass based on values from the sampled posterior distribution. For behavioral data, we computed 97% percentile confidence intervals using non-parametric bootstrapping, 2,000 iterations (Davison & Hinkley, 1997). Data were sampled with replacement across all participants, averages were computed for the relevant combination of conditions per participant and then the group average for the sample was computed. The distribution of bootstrapped samples of group averages was then used to compute 97% percentile confidence intervals.

The fitted models were compared using a leave-one-out (LOO) information criterion (Vehtari et al., 2017). LOO computes an expected log predicted density (ELPD) that expresses the expected out-of-sample deviance based on the posterior distribution of in-sample deviance; see Vehtari et al. (2017) for details. The LOO information criterion is interpreted in the same way as other information criteria, such as Akaike or Widely Applicable Information Criteria (AIC and WAIC, respectively), with lower values indicating better goodness-of-fit given the penalty for model complexity. We reported the difference in expected log predicted density (ΔELPD, mean ± standard error) relative to the best model (top model in each table, ΔELPD = 0). Models in each table are ordered from best to worst based on their ΔELPD relative to the best model. In addition, we used ELPD to compute a relative weight for each model. For this, all models are used as an ensemble to maximize accuracy of leave-one-out cross-validation performance. The overall prediction is computed as a weighted mean of predictions. The weights add up to 1 and higher weights mean a larger contribution to the prediction and, therefore, higher relevance of the individual model. Note that this approach means that higher weights are assigned to models that contain unique additional information, so while the best model tends to have the highest weight, this may not be true for other models. As the direct comparison via ΔELPD and the indirect one via the weights approach provide different model rankings, we presented both.

### Model: The general design of ideal observer models

Our experimental procedure can potentially produce simultaneous priming at three levels: (1) for shapes of individual game objects, (2) for individual colors, and (3) for the attentional sets themselves. Each of these effects reflects the prior history of the game, and the response times are likely to reflect a complex interaction between them, making a simple factorial linear model ineffective for understanding the results. To address this, we opted to design a series of ideal-observer models that could include multiple independent history traces based on a first-order memory process formalization (see below).

The ideal observer models followed the same template with three consecutive processing stages but different processing mechanisms. Specifically, an ideal observer first identifies the attentional set (${RT}_{set}$). Note that this stage is relevant only for the mixed blocks, as participants did not need to identify the attentional set for direct-only and indirect-only blocks. Next, they identified the target object given the attentional set (${RT}_{object}$) and finally, observers needed time to carry out the actual response, which includes motor planning and execution (${RT}_{motor}$). Accordingly, the total response time was computed as:

1 $$RT={RT}_{set}\left(set\right)+{RT}_{object}\left(set\right)+{RT}_{motor}$$

Note that the first two components can depend on the attentional set. For example, the time needed to identify the attentional set may depend on the set itself or the prior history of attentional sets. Similarly, the processing time required to identify the target object is likely to differ between attentional sets. In contrast, the latter component – motor planning and execution – is independent of the attentional set as it is associated with actions occurring after the set and the target object have been identified. In the actual implementation of various ideal observers, ${RT}_{set}$ and ${RT}_{object}$ included their own set-specific intercept terms, e.g.:

2 $${RT}_{object}\left(set\right)={\alpha }_{object}\left(set\right)+{\beta }_{object}\left(set\right)\cdot (1-{salience}_{obj})$$

However, for the sake of sampling efficiency, we opted to sample the total sum of all intercepts of all relevant terms (${RT}_{set}, R{T}_{object},R{T}_{motor}$) instead of including individual terms that sum up to it. In cases like these, the estimated value of ${RT}_{motor}$ does not correspond to the final component alone but to the total sum of relevant intercepts:

3 $${RT}_{motor}={RT}_{set}+{RT}_{object}+{RT}_{motor}^{true}$$

and, therefore, its values cannot be compared across models.

The mechanisms implemented for different stages were independent and we constructed numerous ideal observers using different combinations. To simplify the identification of individual ideal observer models in the repository, we used the following scheme for filenames:

$${[\mathbf{s}\mathbf{e}\mathbf{t}-...]}_{\_}{[\mathbf{o}\mathbf{b}\mathbf{j}-\dots ]}_{\_}[\mathbf{c}\mathbf{o}\mathbf{l}\mathbf{o}\mathbf{r}-\dots ].\mathbf{s}\mathbf{t}\mathbf{a}\mathbf{n}$$

where each bracket part identified mechanisms used to implement the influence of (changing) attentional set, target object, and its color. The former component determines the computation for ${RT}_{set}$, whereas the two latter components jointly determine ${RT}_{object}$.

There are two aspects of the task that influence response times for the *medium* and *hard* difficulty conditions. First, a memory for location of the target object could lead to a location-specific priming effect (Campana & Casco, 2009; Maljkovic & Nakayama, 1996). However, in our experimental design, location has less relevance than in a conventional visual search task. First, it has no meaning for the *easy* difficulty condition as there is only a single object and therefore a single position for the trial set. Second, it has no meaning for the indirect attentional sets as the target is *not* in the trial set, so there is no position that is specific to the target. Nonetheless, we tested this possibility by creating a version of the best performing model for *medium* and *hard* difficulty conditions that included memory for each position within the trial set. This model used the same first-order memory mechanisms as other components (see below), increasing location salience if it contained response object and decreasing location salience for non-targets. A comparison via LOO information criterion (see below, the same approach as we used for comparing all models in the article) showed that the added complexity does not lead to substantial improvement in out-of-sample predictions. Therefore, we included the model in the repository (see Open Practices Statement) but given the limited effect of position and complexity of modelling, we opted not to discuss that particular component in the article.

Similarly, for the *medium* and *hard* difficulty conditions, there was always a uniquely colored object within a game set, a property that could make it more salient. Our analysis confirmed that although the accuracy of responses was unaffected, the response times were indeed faster when this object was a target. We created an additional model that included additional strength modulation for a uniquely colored object, which improved predictions of the model. However, the improvements were moderate, and the factor itself was static, producing no further insight into the priming that the current article focuses on. Therefore, we opted not to discuss this effect further, but to include the model in the repository (see Open Practices Statement).

All models were fitted to empirical data using random effects for all relevant parameters. In other words, every parameter in the model had a group average and distribution components plus individual values for each participant sampled from this distribution. Note that we used mathematically equivalent non-centered parameterization to facilitate sampling from multivariate distributions.

### Model: First-order memory process

For the ideal observer model described below, we assumed that all memory traces evolved as a first-order process, with their strength increasing whenever a corresponding attentional set, object, or color were present on the current trial and decreasing whenever they were absent. The general formulation is:

4 $${M}^{x}\left(t\right)={M}_{0}^{x}{e}^{-t/\tau }$$

In our case, we have a time step of 1 (trial) and, therefore, we fixed the upper boundary at 1 (if x is present) and the lower boundary at 0 (if x is absent). Thus, the discrete version of the same process is:

5 $${M}^{x}\left[t\right]=B[t]+\left({M}^{X}\left[t-1\right]-B[t]\right)\cdot {e}^{-\frac{1}{\tau }}$$

or equivalently:

6 $${M}^{x}\left[t\right]={M}^{X}\left[t-1\right]+\left(B\left[t\right]-{M}^{X}\left[t-1\right]\right)\cdot \left(1-{e}^{-\frac{1}{\tau }}\right)$$

where:

7 $$B\left[t\right]=\left\{\begin{array}{cc}1& if\, x\, is\, present\\ 0& if\, x\,is\, absent\end{array}\right.,$$

and *x* is an attentional set, object, or color.

We assumed that all traces (for attentional set, object, and color) are in a neutral state at the beginning of each block:

8 $${M}^{X}\left[0\right]=0.5$$

Considering that the proportion constant is $0\le \left(1-{e}^{-\frac{1}{\tau }}\right)\le 1$ for $\tau \ge 0$, we opted to sample the entire expression rather than the $\tau$ itself. Accordingly, the proportion constant per participant was:

9 $$logit\left(1-{e}^{-\frac{1}{\tau }}\right) \sim Normal\left({\mu }^{\tau }, {\sigma }^{\tau }\right),$$

10 $${\mu }^{\tau }\sim Normal\left(0, 1\right),$$

11 $${\sigma }^{\tau }\sim Exponential(10)$$

**Fig. 3:**
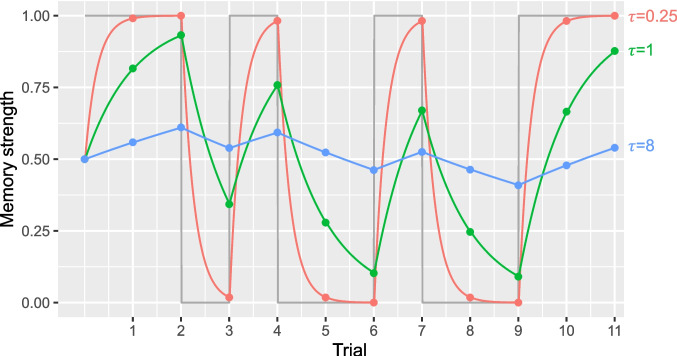
Evolution of memory as a first-order process for different time constants. The gray dashed line indicates presence (1) or absence (0) of a target during the trial. The colored lines and circles indicate memory strength as a function of time and target presence for three different time constants, τ

Examples of memory evolution for different time constants are depicted in Fig. 3. Note that the time constants control how quickly the memory builds up and, therefore, the memory span. This means that larger time constants lead to slower evolution of the memory and reflect a longer history interval (τ = 8 in Fig. 3, τ = ∞ means constant memory state and, therefore, no memory). Conversely, shorter time constants lead to faster-evolving memory with a shorter history span. For instance, for τ = 0.25, memory traces become almost 1 after two repetitions of the target. In the extreme case of τ = 0, the only information contained in the memory is about the most recent trial only, as that target memory strength becomes 1 and that of all distractors 0.

We also assumed that the conspicuousness of a given object or set is computed via divisive normalization. For example, for attentional sets, this means that the conspicuousness of a direct attentional set is computed by dividing its memory trace strength by the sum of memory traces’ strengths for both direct and indirect sets. Thus, the conspicuousness of the direct set is relative to the sum of memories of both sets.

12 $${S}_{j}^{x}=\frac{{M}_{j}^{x}}{\sum_{i=1}^{{N}^{x}}{M}_{i}^{x}}$$

When several memory traces were used in an ideal observer model, we sampled the time constant for each memory from a correlated multivariate Gaussian distribution to facilitate sampling convergence. For example, when an ideal observer model had separate time constants for memory of attentional sets and objects, they were sampled from a bivariate Gaussian using the same priors for μ and σ as the univariate Gaussian above but with the additional correlation term *R*.

13 $$\left[\begin{array}{c}\mathrm{logit}\left({e}^{-1/{\tau }_{set}}\right)\\ \mathrm{logit}\left({e}^{-1/{\tau }_{object}}\right)\end{array}\right]\sim MVNormal\left(\left[\begin{array}{c}{\mu}_{set}^{\tau}\\ {\mu}_{object}^{\tau }\end{array}\right], {S}_{\tau }\right),$$

14 $${S}_{\tau }=\left(\begin{array}{lc}{\sigma}_{set}^{\tau}\ \ \ \ \ \ \ \ 0\\ 0\ \ \ \ \ \ \ \ \ \ \ {\sigma}_{object}^{\tau }\end{array}\right){R}_{set}\left(\begin{array}{lc}{\sigma }_{set}^{\tau }\ \ \ \ \ \ \ \ 0\\ 0\ \ \ \ \ \ \ \ \ \ \ {\sigma }_{object}^{\tau}\end{array}\right),$$

we refer to this process as ${f}_{m1}(\tau)$ in the text below.

### Model component: Time required to identify attentional set during mixed blocks, ${{\boldsymbol{R}}{\boldsymbol{T}}}_{{\boldsymbol{s}}{\boldsymbol{e}}{\boldsymbol{t}}}$

Below is the description of mechanisms that define the extra processing time required to identify an attentional set during mixed blocks: ${RT}_{set}$. Each mechanism is identified using the **[set_...]** label in the model filename. Icons are used to identify model components in figures; they are summarized also in Table 3.

**Table 3:**
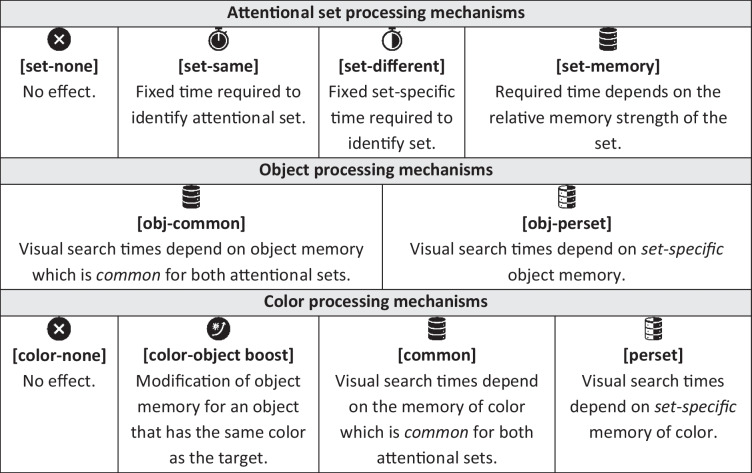
Ideal observer components used in the modular design. Icons identify components in figures below, bold text in brackets shows how the component was labeled within the filename in the online repository. Note that color-processing mechanisms were irrelevant for the *easy* difficulty condition, where all game objects had a unique color. See *Model* section for details on the implementation of individual mechanisms

**[set-none]** The simplest, if unrealistic, mechanism used for baseline comparison. Here, an ideal observer required no time to identify the attentional set even during mixed blocks:

15 $${RT}_{set}=0$$

** [set-same]** An ideal observer required the same amount of time to identify the set, irrespective of which attentional set was used during the trial. ${RT}_{set}$ was sampled per participant from a normal distribution with a log link with priors centered at 0.02 s, i.e., we used conservative regularizing priors that favored brief processing times:

16 $$\mathrm{log}\left({RT}_{set}\right)\sim Normal\left({\mu }_{set},{\sigma }_{set}\right),$$

17 $${\mu }_{set}\sim \mathrm{Normal}(\mathrm{log}\left(0.02\right), 2),$$

18 $${\sigma }_{set}\sim Exponential(1)$$

** [set-different]** An ideal observer model similar to the one above but assumed that processing time was set-specific, i.e., one attentional set was identified quicker than the other. Response times were sampled from a bivariate Gaussian with a log link. We assumed that costs for identifying the set were correlated. Intuitively, participants who were faster than average at identifying the direct attentional set could also be faster than average at identifying the indirect one. However, the formulation allows equally for a negative and zero correlation.

19 $$\left[\begin{array}{c}\mathrm{log}\left({RT}_{set}^{direct}\right)\\ \mathrm{log}\left({RT}_{set}^{indirect}\right)\end{array}\right]\sim MVNormal\left(\left[\begin{array}{c}{\mu }_{set}^{direct}\\ {\mu }_{set}^{indirect}\end{array}\right], {\Sigma }_{set}\right),$$

20 $${\Sigma }_{set}=\left(\begin{array}{cc}{\sigma }_{set}^{direct}& 0\\ 0& {\sigma }_{set}^{indirect}\end{array}\right){R}_{set}\left(\begin{array}{cc}{\sigma }_{set}^{direct}& 0\\ 0& {\sigma }_{set}^{indirect}\end{array}\right),$$

21 $${\mu }_{set}^{direct}, {\mu }_{set}^{indirect} \sim Normal\left(\mathrm{log}\left(0.02\right), 2\right),$$

22 $${\sigma }_{set}^{direct}, {\sigma }_{set}^{indirect}\sim Exp\left(10\right),$$

23 $${R}_{set}\sim \mathrm{LKJcorr}(4)$$

** [set-memory]** Here, ${RT}_{set}$ was modulated by the relative strength of the trial attentional set (${S}_{set}^{direct}$ and ${S}_{set}^{indirect}$) that reflected the prior history of attentional sets within the block. We assumed that the memory of an attentional set (${M}_{set}^{direct}$ and ${M}_{set}^{indirect}$) evolves as a first-order process (see above) so that:

24 $$\begin{array}{c}{M}_{set}^{direct}\\ {M}_{set}^{indirect}\end{array}\sim {f}_{m1}({\tau }_{set})$$

and

25 $${S}_{set}^{direct}=\frac{{M}_{set}^{direct}}{{M}_{set}^{direct}+{M}_{set}^{indirect}}$$

26 $${S}_{set}^{indirect}=\frac{{M}_{set}^{indirect}}{{M}_{set}^{direct}+{M}_{set}^{indirect}}$$

The actual response times were computed per set as:

27 $${\alpha }_{\mathrm{set}}^{\mathrm{direct}}+\mathrm{log}\left({RT}_{set}^{direct}\right)\cdot (1- {S}_{set}^{direct}\left[t\right]),$$

28 $${\alpha}_{\mathrm{set}}^{\mathrm{indirect}}+\mathrm{log}\left({RT}_{set}^{indirect}\right)\cdot {(1- S}_{set}^{indirect}\left[t\right]),$$

i.e., stronger memory traces led to shorter response times and vice versa.

$\mathrm{log}\left({RT}_{set}^{direct}\right)$ and $\mathrm{log}\left({RT}_{set}^{indirect}\right)$ were modeled as in **[set-different].** ${\alpha}_{\mathrm{set}}^{\mathrm{direct}}$ and ${\alpha}_{\mathrm{set}}^{\mathrm{indirect}}$ were not modeled explicitly but were incorporated into a set-specific ${RT}_{motor}$ (see reasoning above).

### Model component: Time required to identify the target object, ${{\boldsymbol{R}}{\boldsymbol{T}}}_{{\boldsymbol{o}}{\boldsymbol{b}}{\boldsymbol{j}}}$

We assumed that search times for the target object reflect its relative strength of representation (${S}_{obj}$), which reflected:

1. Influence of prior history of this object being a target object during previous trials.
2. Influence due to the conspicuousness of its color.
3. Additional modulation due to being present in the set.

Note that the latter two components were relevant only for the *medium* and *hard* difficulty conditions, as for the *easy* difficulty condition all objects had unique colors and an attentional set consisted only of a single object.

The processing time was computed as:

29 $${\alpha }_{obj}^{direct}+\mathrm{log}\left({RT}_{obj}^{direct}\right)\cdot (1- {S}_{obj}),$$

30 $${\alpha }_{\mathit{obj}}^{\mathit{indirect}}+\mathrm{log}\left({RT}_{obj}^{indirect}\right)\cdot (1- {S}_{obj}),$$

where ${\alpha}_{\mathrm{obj}}^{\mathrm{direct}}$ and ${\alpha}_{\mathrm{obj}}^{\mathrm{indirect}}$ were not modeled explicitly but were incorporated into a set-specific ${RT}_{motor}$ (see reasoning above). The strength for a given object ${S}_{obj}[i]$ was computed via divisive normalization of context-modulated memory traces (${M^{^\prime}}_{obj}$):

31 $${S}_{obj}\left[i\right]=\frac{{M^{^\prime}}_{obj}\left[i\right]}{\sum\nolimits_{j}{M^{^\prime}}_{obj}[j]}$$

The context effects of presence and color (${w}_{context}$) produced a non-linear modulation based on the strength of the memory itself (${M}_{obj}$):

32 $${M^{^\prime}}_{obj}={M}_{obj}+\left(1-{M}_{obj}\right)\cdot {w}_{context}$$

The context effect is defined as modulation due to the object being present in the set and the influence of the color memory. The former depends on the strength of modulation (modulation weight ${w}_{presence}$) and an indicator variable $Present\left[t\right]$ that indicates the presence (1) or absence (0) of the object in the attentional set. The influence of the color memory is modulated by the corresponding weight (${w}_{color}$) and the strength of the color memory trace at time t (${M}_{color}\left[t\right]$, see below for details):

33 $${w}_{context}={w}_{presence}\cdot Present[t]+{w}_{color}\cdot {M}_{color}$$

Please note that context modulation was a subject to constrain $0\le {w}_{context}\le 1$, i.e., the value of ${w}_{context}$ was clipped to the range of [0..1].

With respect to the influence of prior history ${M}_{obj}$, ideal observers utilize a memory trace evolving as a first-order process (see above) but differ in whether there was a single memory, common for both attentional sets, or two object memory representations, one per set.

** [obj-common]** A memory representation was independent of the attentional set, i.e., common for both.

34 $${M}_{object}\sim {f}_{m1}({\tau }_{object})$$

** [obj-perset]** Two set-specific memory representations.

35 $${M}_{object}^{direct}\sim {f}_{m1}\left({\tau }_{object}\right)$$

36 $${M}_{object}^{indirect}\sim {f}_{m1}({\tau}_{object})$$

The effect of being present in the attentional set ${w}_{presence}$ and modulation due to color ${w}_{color}$ were both sampled per attentional set and per participant from a bivariate distribution. The group prior was centered at approximately 0.1 modulation. We describe the model for ${w}_{presence}$ but an identical model was implemented for ${w}_{color}$ (modulation due to the strength of the color memory trace).

37 $$\left[\begin{array}{c}\mathrm{logit}\left({w}_{presence}^{direct}\right)\\ \mathrm{logit}\left({w}_{presence}^{indirect}\right)\end{array}\right]\sim MVNormal\left(\left[\begin{array}{c}{\mu }_{presence}^{direct}\\ {\mu }_{presence}^{indirect}\end{array}\right], {\Sigma }_{presence}\right),$$

38 $${\Sigma }_{precense}=\left(\begin{array}{cc}{\sigma }_{presence}^{direct}& 0\\ 0& {\sigma }_{presence}^{indirect}\end{array}\right){R}_{presence}\left(\begin{array}{cc}{\sigma }_{presence}^{direct}& 0\\ 0& {\sigma }_{presence}^{indirect}\end{array}\right),$$

39 $${\mu }_{presence}^{direct}, {\mu }_{presence}^{indirect} \sim Normal\left(-2, 2\right),$$

40 $${\sigma }_{presence}^{direct}, {\sigma }_{presence}^{indirect}\sim Exp\left(1\right),$$

41 $${R}_{presence}\sim \mathrm{LKJcorr}(4)$$

With respect to the influence of the color, we implemented four different mechanisms.

**[color-none]** The simplest algorithm that assumed no influence of color on response times, i.e., the term ${w}_{color}\cdot {M}_{color}\left[t\right]$ was omitted when computing object strength (${S}_{obj}$).

** [color-object boost]** Here, we assumed that color did not have a separate memory trace but memory traces for an object that had the same color as the target were adjusted upwards in proportion to the adjustment of the target object itself. Specifically, the proportionality constant was adjusted as

$${w}_{color}\cdot \left(1-{e}^{-\frac{1}{\tau }}\right)$$

See details on the first-order memory process above, where $0\le {w}_{color}\le 1$.

** [color-common]** This algorithm assumed a common memory for colors.

42 $${M}_{color}\sim {f}_{m1}({\tau }_{color})$$

The relative strength of the color was computed via a divisive normalization, identical to that used for set and object memory normalization.

** [color-perset]** This algorithm assumed two memories for colors, one per attentional set.

43 $$\begin{array}{c}{S}_{color}^{direct}\\ {S}_{color}^{indirect}\end{array}\sim {f}_{m1}({\tau }_{color})$$

### Model component: Time required for carrying out the response, ${{\boldsymbol{R}}{\boldsymbol{T}}}_{{\boldsymbol{m}}{\boldsymbol{o}}{\boldsymbol{t}}{\boldsymbol{o}}{\boldsymbol{r}}}$

As noted above, ${RT}_{motor}$ should not depend on the attentional set as it reflects motor planning and execution only after both the attentional set and the target object were identified. However, for reasons of sampling efficiency, it absorbed set-specific intercept terms from ${RT}_{set}$ and ${RT}_{object}$. Therefore, ${RT}_{motor}$ was sampled per attentional set and participant from a bivariate Gaussian with a log link. We assumed that the total sum of intercepts was likely to be correlated between the sets. We used identical weakly regularizing priors for the mean-centered at 0.6 s and strongly regularizing priors for variance to facilitate sampling.

44 $$\left[\begin{array}{lc}\mathrm{log}\left({RT}_{motor}^{direct}\right)\\ \mathrm{log}\left({RT}_{motor}^{indirect}\right)\end{array}\right]\sim MVNormal\left(\left[\begin{array}{lc}{\mu}_{motor}^{direct}\\ {\mu}_{motor}^{indirect}\end{array}\right], \sum\nolimits_{motor}\right),$$

45 $$\sum\nolimits _{motor}=\left(\begin{array}{lc}{\sigma}_{motor}^{direct}\ \ \ \ \ 0\\ 0\ \ \ \ \ \ \ \ \ \ \ {\sigma}_{motor}^{indirect}\end{array}\right){R}_{motor}\left(\begin{array}{lc}{\sigma}_{motor}^{direct}\ \ \ \ \ \ \ 0\\ 0 \ \ \ \ \ \ \ \ \ \ \ \ \ {\sigma}_{motor}^{indirect}\end{array}\right),$$

46 $${\mu }_{motor}^{direct}, {\mu}_{motor}^{indirect} \sim Normal\left(\mathrm{log}\left(0.6\right), 1\right),$$

47 $${\sigma}_{motor}^{direct}, {\sigma}_{motor}^{indirect}\sim Exp\left(10\right),$$

48 $${R}_{motor}\sim \mathrm{LKJcorr}(4)$$

## Results

We investigated how attentional sets and prior history influence the speed of responses in a target identification task. In the experiment, players must identify a target among the game objects based on objects present in the attentional set for a given trial. Crucially, the attentional set can be direct when the target object is the only object within the set depicted in the correct color and, conversely, an attentional set is indirect when objects and colors in the set serve as exclusion criteria to identify a unique remaining target object. The task varied in difficulty on three levels, based on the number of game objects, colors, and objects in the set: (i) *Easy* (three game objects, three colors, one object in the attentional set); (ii) *medium* (five objects, three colors, and two objects in the set); and (iii) *hard* (nine objects, five colors, and three objects in the set). See the *Experimental procedure* section and Fig. 1 in *Materials and* *methods* for further details, illustrations, and example trials.

**Fig. 4:**
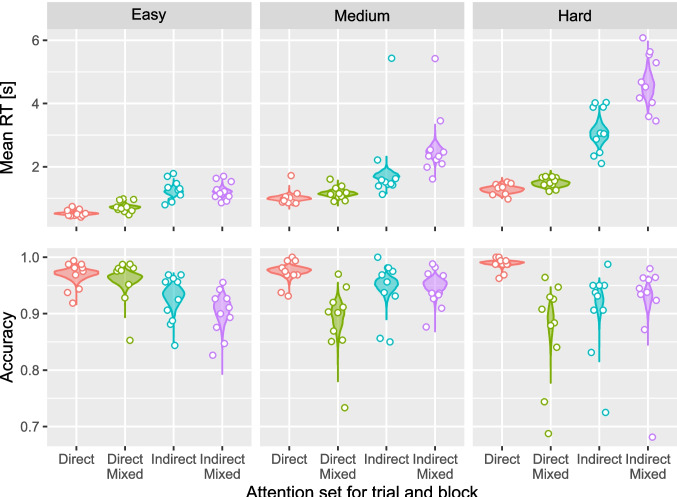
Response time (**top row**) and accuracy (**bottom row**) across the three difficulty conditions as a function of the four different conditions (direct set only blocks; direct set within mixed trial blocks; indirect set only blocks; indirect set within mixed trial blocks). Circles depict individual participants (geometric mean for individual response times), and violin plots show group-level posterior predictions of the multilevel Bayesian model. The chance level is 1/3, 1/5, and 1/9 for *easy*, *medium*, and *hard* difficulty, respectively

The overall accuracy and response times are shown in Fig. 4. Higher difficulty (larger game objects set) led in all cases to longer response times (> 98% of samples show a positive difference in posterior predictions at the group level). Similarly, mixing attentional sets within a block led to longer response times than in blocks with fixed attentional sets (> 99% of samples show a positive difference in posterior predictions at the group level apart from indirect-only vs. indirect-mixed conditions for *easy* difficulty where differences are not consistently positive and, therefore, not significant). In contrast, there was no significant change in accuracy as a function of difficulty level (a complete comparison table is available in the online repository).

Mixing attentional sets within a single block had a strong effect on accuracy on trials with the direct attentional set (highly significant for *medium* and *hard* difficulty, see accuracy plot in Fig. 4) but little or no effect on indirect set trials. Moreover, accuracy was, in general, the poorest for the direct attentional set on mixed block trials for *medium* and *hard* difficulty. We surmise that this reflects that observers become stuck on applying the indirect set in an attempt to find a plausible-looking exclusion-based target only later realizing that this was a direct-set trial.

The response times showed the same pattern across all experiments with mixed blocks producing significantly lower response times for both direct and indirect attentional sets (see Response times plot in Fig. 4). We sought to quantify these differences by measuring priming effects, as our experimental design can potentially lead to simultaneous priming at three levels:

1. For individual game objects,
2. For individual colors, and
3. For the attentional sets themselves.

Although trials were fully randomized, one approach to analyzing such data is to identify sequences of trials with repetitions, computing priming effects and fitting them using generalized linear models to estimate the processing costs (response times) and their reduction due to repetition. However, there are multiple possible combinations with repetitions at a single level (e.g., repetition of an attentional set but changing target object and color), at two levels (e.g., repetition of object and color but not of attentional set), or all three levels (repetition of object, color, and attentional set). All these priming effects likely reflect interplays between processing stages and should, therefore, reflect predictions of a single model rather than those of multiple repetition-combination-specific models.

**Fig. 5:**
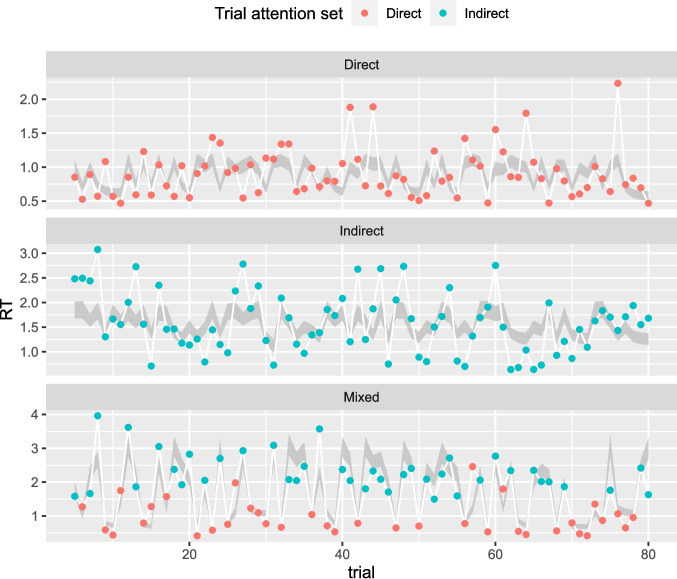
Example time-series for a demonstrative participant in *medium* difficulty condition. Time series for the last three blocks. Circles and white lines depict actual response times, color denotes the attentional set on a given trial (red – direct, blue – indirect). The gray stripe corresponds to a 97% confidence interval for posterior predictions of a model used to evaluate repetition priming (see below)

Our solution was to develop a set of ideal observer models with a modular design where we sought to predict response times for individual trials rather than the repetition priming effects themselves. Therefore, each model was used to predict *all* response times throughout the experiment, irrespective of whether they would be used for later plotting and comparison. An example of such a time series for the best performing model for *medium* difficulty (see below) overlayed with actual response times is shown in Fig. 5. Our approach means that any repetition priming effects are an emergent property of the assumed mechanisms, revealing which mechanisms replicate the performance of real participants. However, we do not claim that our best-performing models accurately reflect actual neural computation and networks. In fact, below we show that their performance depends on the context, highlighting the need for further research and development.

The ideal observer models used a modular approach, assuming that the decision-making process proceeds through three stages. Firstly, players needed to identify the attentional set (direct or indirect). Secondly, they needed to identify the target object, conditional on the attentional set and objects present within it. Finally, they needed to respond. We assumed the last part to be constant over participants and concentrated on mechanisms reflecting processing of the prior history of attentional sets, target objects, and color. For each stage, we formulate several different possible mechanisms (see Table 3 and *Methods* for details). Therefore, we coded each mechanism for every representation and fitted different combinations to the actual response times of all real players (Fig. 5). We used a pooled-parameter random effect approach so that ideal observer models for individual participants were structurally identical but differed in parameter values that were sampled from group-level parameter distribution. This allowed estimates at both group and individual levels.

**Fig. 6:**
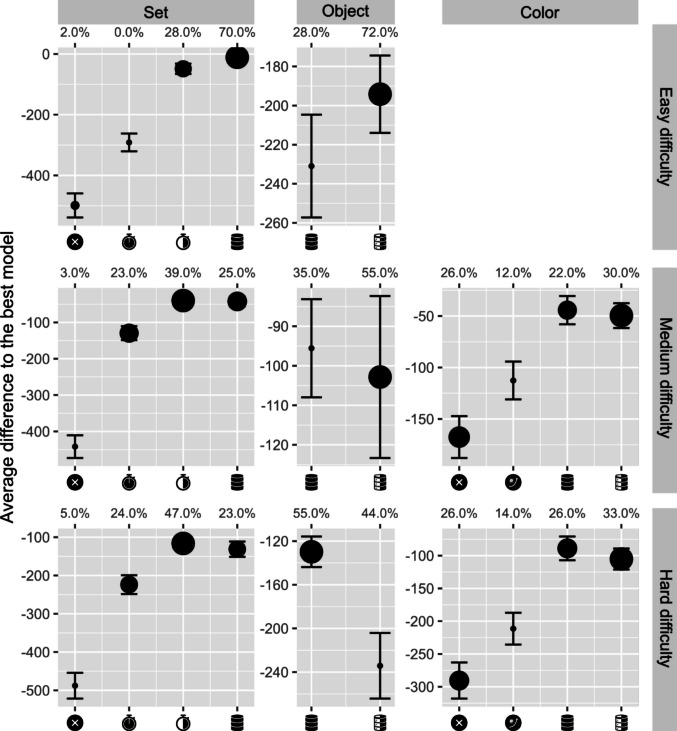
Relative performance of models for each difficulty (rows) and stage (columns) given a specific stage component. X-axis labels encode component, see Table 3 for details. The Y-axes show average ΔELPD and its average error relative to the best model across all models that the component was part of; higher values mean better out-of-sample predictions. Circle size and text above the plot show the total weight of all models the component was part of

We compared the predictive performance of the ideal observer models using the LOO information criterion (Vehtari et al., 2017) that estimates accuracy of LOO cross-validation, i.e., the model’s expected performance on out-of-sample data points (ΔELPD) and their weight as part of a linear combination of models that maximizes cross-validation performance (Weight), see *Statistical analysis* section in *Methods*. Summaries of these comparisons are presented in Figs. S1–S3 in the Online Supplementary Material. We were not interested in specific models but instead in individual components for each processing stage that better predicted performance across *all* models. For this, we computed average ΔELPD and the total weight of all models that a given component was a part of (Fig. 6). Note that well estimated out-of-sample performance does not necessarily mean that we correctly identified exact mechanisms for a specific component, merely that they are the best among those tested. For example, for both the *medium* and the *hard* difficulty conditions, models with independent color memory representations per attentional set have higher weight than the rest. However, this does not necessarily mean that there are two actual memory traces, merely that this is the best option given the structural constraints of the model. At the same time, this shows that the influence of color probably qualitatively differs between two attentional sets, so any future models and research should consider this.

When we consider *all trials* across *all attentional* *sets*, repetitions of sets, objects, and colors (Fig. 6), our results are consistent with a memory trace that tracks attentional sets and is used to identify them on the current trial. A simple response time difference could explain the results, but its simpler structure is offset by poorer predictive performance, making two solutions comparable, hinting at the contribution of memory mechanisms. With respect to game objects, a single memory trace is sufficient to explain response times across both attentional sets, as the assumption of two independent traces drastically changes predictive performance. However, as noted above, color repetition could rely on different mechanisms for the two attentional sets.

**Fig. 7:**
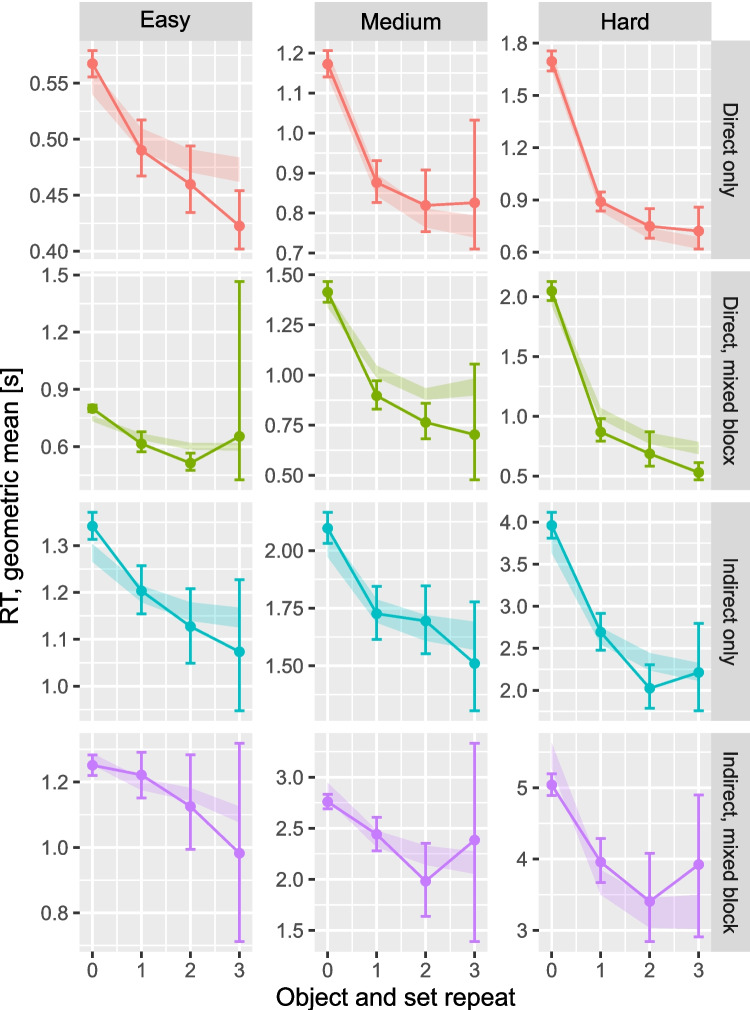
Effect of repetition of the attentional set and target object for each difficulty (columns), set and context (rows). Lines and error bars depict the group average and 97% non-parametric bootstrapped confidence intervals for real players (2,000 iterations). Stripes show 97% compatibility/credible intervals for posterior predictions of the best model for the difficulty condition at the group level. See Online Supplementary Material, Tables S1, S4, and S8

The comparison in Fig. 6 is across *all* trials, but our main interest is how well different models reproduce the various repetition effects. To this end, we selected different trial subsets and compared the emergent pattern for real participants and ideal observer models. The simplest case is a repetition of an attentional set, target object, and target color (the latter repetition is trivial due to repetition of the target object) as presented in Fig. 7; this shows both strong repetition priming for human participants and quantitively similar repetition priming in the ideal observer models for both direct and indirect attentional sets.

**Fig. 8:**
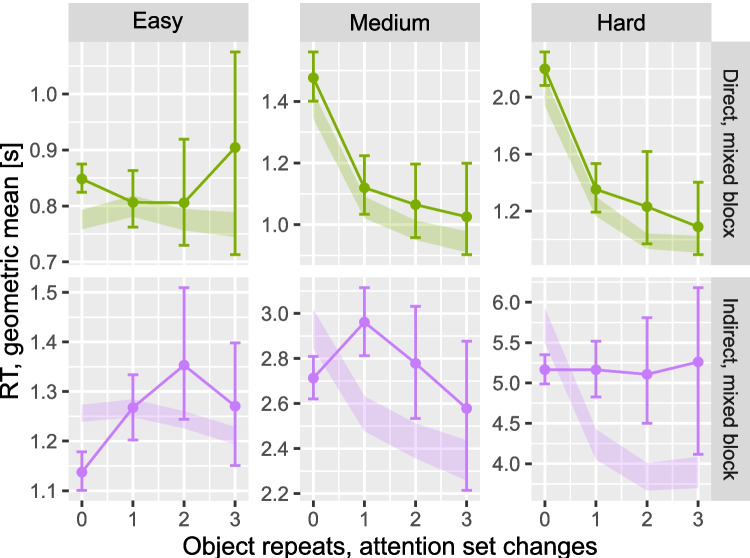
Effect of repetition of target object while attentional set changes. See the caption of Fig. 7 for details. See Online Supplementary Material, Tables S2, S5, and S9

However, the limitations of our ideal observer models become quite evident when we consider other repetition patterns. For example, when a target object repeats but the attentional set changes (Fig. 8), human participants show only a priming effect for the direct but not for the indirect attentional sets, something the ideal observer model fails to capture – exhibiting instead repetition priming for indirect sets.

**Fig. 9:**
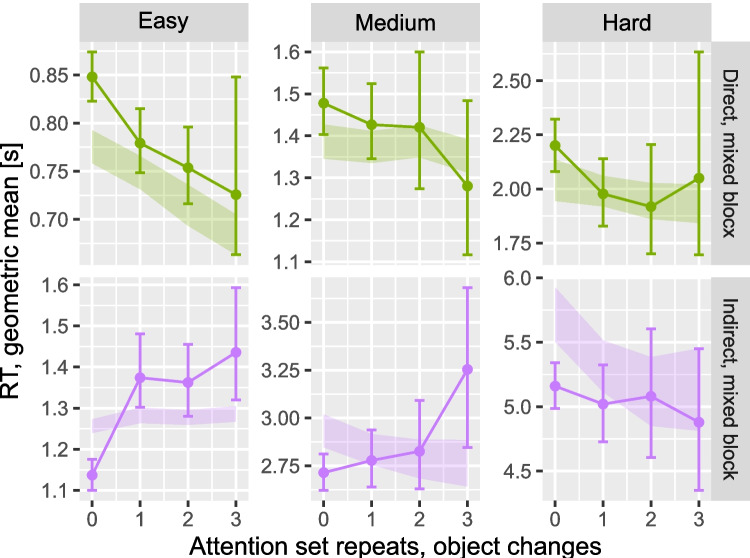
Effect of repetition of the attentional set while target object changes. See the caption of Fig. 7 for details. See also Online Supplementary Material, Tables S3, S6, and S10

Similarly, when the attentional set repeats but the target object changes (Fig. 9), human performance shows positive priming for the direct set but negative priming for the indirect sets, which the ideal observer models are again unable to capture

**Fig. 10:**
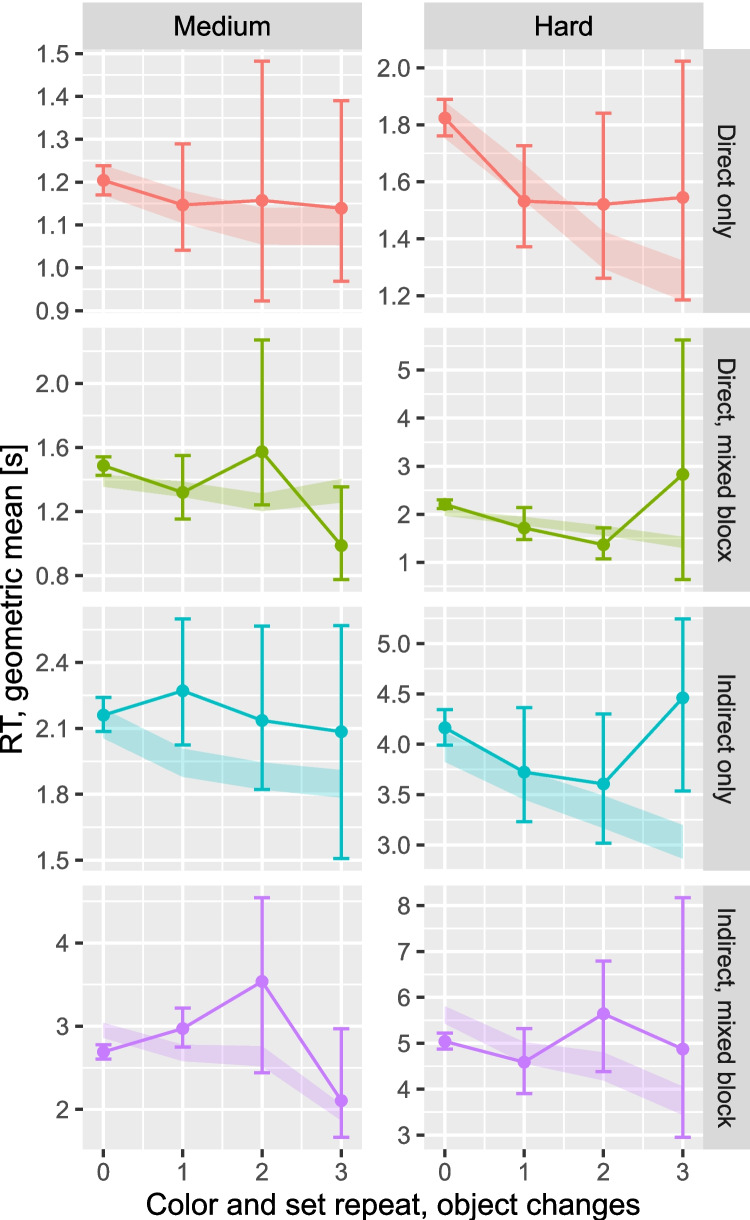
Effect of repetition of target color and attentional set while target object changes. See the caption of Fig. 7 for details. See also Online Supplementary Material, Tables S7 and S11

Finally, there are no clear patterns for human participants for the model when color and attentional set repeat across trials, but not the target object (Fig. 10), an interesting result since it indicates what the limits are and what sort of information transfers between trials. What might explain that priming is not seen when attentional set and color repeat, but the object is not? One clue is the observation of Ásgeirsson and Kristjánsson (2011) who found that the signal strength of target against distractors had a strong effect on the manifestations of priming. During difficult search with low saliency of targets versus distractors, priming was episodic, while when the feature contrasts between target and distractors were increased, priming of different features was independent. We suspect something akin to the low-saliency situation may be at play here.

**Fig. 11:**
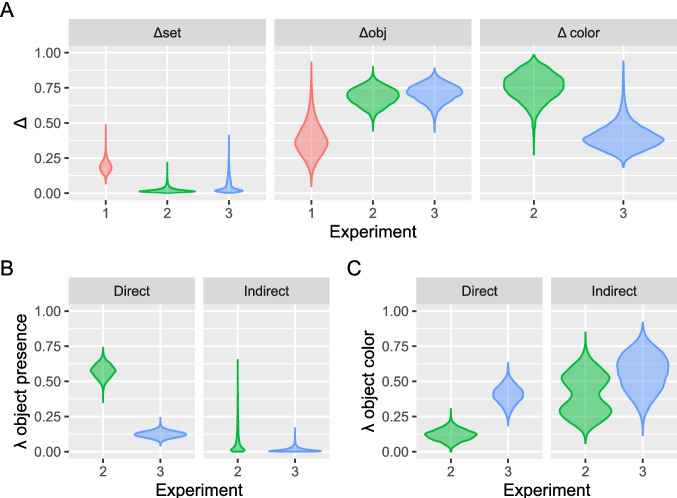
The posterior distribution for population-level parameters for a selected subset of models. Following the leave-one-out analysis above, these included memory mechanisms for attentional set and distinct memory traces for color. Plots for all population-level posterior distributions are available in the online repository. (**A**) Memory-adjustment proportion parameter Δ for attentional set, object, and color memory traces. (**B**) Additional modulation for an object’s presence in the set. (**C**) Additional modulation from object color

The posterior distributions for memory time constants are very different between the attentional set and object/color memory (Fig. 11A). In the former case, there was only a small trial-to-trial change consistent with a comparable performance of models without attentional set memory. In other words, for most ideal observer models, response times were only weakly modulated by attentional set history. In contrast, large update constants for both object and color traces show fairly short-term memory, for example, an update constant of 0.5–0.75 translates into memory that reflects the last three to five trials.

The analysis of relative object salience due to its presence in the set shows qualitative differences between direct and indirect attentional sets (Fig. 11B). Namely, the presence of a non-target object in the set increases its salience, interfering with the search for a target in the direct set. However, there is no interference for indirect attentional sets. This is consistent with the fact that in this case in-set objects can act as cues, not as competing distractors, and facilitate rather than hinder the search since the target is defined by exclusion of colors and objects. Finally, color modulated performance with a stronger effect for the indirect than direct attentional set (Fig. 11C).

## Discussion

Our aim was to cast a light on different ways of tuning attentional sets. While a lot of research is devoted to the direct set, how observers search for a prespecified target, much less research is devoted to the converse case (indirect sets), where the item that observers search for is defined by exclusion, and attention cannot be guided to particular colors or shapes. We investigated a task where observers had to find a target either using an attentional set containing the target itself (*direct* set) or an attentional set containing only cues that define the target by exclusion (*indirect* set). The following are the main conclusions of our experiment:

i) Response times differed significantly between attentional sets, with much slower performance for the indirect than the direct cue.
ii) Response times were significantly slower when sets were mixed within blocks, with more time required to identify an indirect than a direct set.
iii) We found strong repetition priming when both set and target were repeated. Other repetition cases led to priming but only for direct attentional sets, with little or no priming observed for indirect attentional sets.
iv) Ideal observer models revealed that for the direct attentional set, emergent repetition priming is consistent with a first-order memory mechanism that tracks objects and colors but not necessarily attentional sets.
v) For the indirect attentional set, our ideal observer models, in most cases, failed to adequately reproduce the results, suggesting that the indirect set reflects the operation of mechanisms and search strategies different from conventional direct attentional set.

In short, our main finding is that the difference between the direct and indirect set is not merely a quantitative one (different response times), but a genuine qualitative one as the two conditions show mostly qualitatively different repetition priming behavior. This is strong evidence that while the direct attention set is likely to rely on the same or similar mechanisms to those involved in a typical visual search, the indirect target search relies on very different processing.

### Differences between direct and indirect attentional sets

When it comes to the overall comparison between the attentional sets, there was a marked difference in observers’ responses for the indirect versus direct set. A likely reason is the larger number of items that participants must process. The direct attentional set is limited to the items visible on the cue card, and, therefore, limited to one (effectively reducing the task to automating the manual response), two, or three items. In contrast, for the indirect search, *all* items must be considered, starting with the ones on the cue card, before elimination reveals the target object. This means 2.5 (Exp. 2) to 3 (Exp. 1 and 3) times more items to search through. This may also reflect a slower mismatch-before-elimination procedure.

Overall, there were strong priming effects when objects were repeated, and in some cases, they were similar across the two attentional sets apart from the slower search times mentioned above (Fig. 7). Interestingly, this priming of objects survives a change to a direct set but disappears with switches to indirect ones (Fig. 8). Similarly, repetition of the attentional set while the target object changes led to decreased response times for the direct attentional set but caused no priming effect (and seemingly resets response times) for the indirect one (Fig. 9). This pronounced sensitivity of the indirect attentional set to changes suggests a less automatic process involving actively maintained working memory, in stark contrast with the robust memory that seems to underpin the direct attentional set priming, (see Magnussen & Greenlee, 1999; Nakayama et al., 2004). For the indirect attentional set, when there are no changes to either the target object or the attentional set, repeating the previous target facilitates response times, but a change to either appears to introduce a discontinuity that resets expectations. A straightforward prediction for future research is that indirect attentional set search should be extremely inefficient in the situation of inattention or when there is interference with working memory in between trials.

The results for the priming of the direct versus indirect set may suggest boundaries on what has often been called “priming of pop-out” but is more accurately referred to as attentional priming. Priming operates on target features (Maljkovic & Nakayama, 1994), objects (Ásgeirsson & Kristjánsson, 2011; Hillstrom, 2000), and locations (Campana & Casco, 2009; Maljkovic & Nakayama, 1996) but our results suggest that the indirect attentional set reflects very different mechanisms from those causing these priming effects.

### Identifying the attentional set

One intriguing question is how participants identify the attentional set itself. One obvious strategy could be to attempt a direct search and if that fails, try an indirect search. However, the observed priming effects (Fig. 7) suggest that this strategy can be overridden by expectations of attentional set repetition, which leads to the curious mistake of searching by exclusion using an indirect attentional set. In such cases, a candidate target object can be located, and the mistake is not apparent until the participant learns that the attentional set is direct. In future research, it would be interesting to find ways of dissociating the timing of the two processes – identification of the set and identification of the target.

Another game that directly addresses attentional selection is the game SET, which has been considerably investigated in the past (Eckstein et al., 2019; Jacob & Hochstein, 2008; Nyamsuren & Taatgen, 2011, 2013). SET shares many features with Geistesblitz, particularly in relation to direct versus indirect attentional set. The selection is less direct in SET than in Geistesblitz, making the latter potentially a better candidate for studying stimulus-driven attentional selection while SET allows the study of grouping and similarity-based perceptual processing, among other things (Jacob & Hochstein, 2008). Previous investigations have suggested that performance in SET involves parallel bottom-up and top-down processing and that considerable priming of strategies occurs between consecutive selections (Nyamsuren & Taatgen, 2011, 2013). Those results have also provided information about how conceptual and perceptual processes interact during attentional selection.

### Ideal observer models

To assess the results, we used multiple ideal observer models which were motivated by the complex interactions between the history of attentional sets, objects, and colors. A simple automatic first-order memory process did a remarkably good job of reproducing repetition priming effects for the direct attentional set in a variety of situations. This suggests that the underlying neural mechanisms, (e.g., Brinkhuis et al., 2020; Campana et al., 2002; Kristjansson et al., 2007) are likely to be based on similar principles and our model provides a good starting point for studying these processes. At the same time, apart from the situation where everything repeats (Fig. 7), our ideal observer failed to capture the lack of repetition priming in most situations for the indirect attentional set. However, this should not be considered a downside to the model per se as it was *designed* to allow for priming and the fact that it was beneficial in *some* situations led to behavior when it also predicted non-existing priming effects elsewhere. This prediction “failure” is informative as it points to either a completely different mechanism or a mixture of mechanisms between low-level and high-level priming mechanisms, the latter possibly involving working memory (see above).

### Connections with probable neural mechanisms

Above, we discussed how our results suggest that a first-order memory mechanism tracks objects and colors but not attentional sets. Evidence from single-cell neurophysiology has shown that neural activity in the so-called frontoparietal attentional network is modulated by feature repetition (Bichot & Schall, 1999, 2002; Westerberg et al., 2020; see Westerberg & Schall, 2021 for review). fMRI studies have revealed that regions within the frontoparietal network show repetition suppression as color priming builds up sequentially over several trial presentations (Becker et al., 2014; Brinkhuis et al., 2020; Geng et al., 2006; Kristjansson et al., 2007; Rorden et al., 2011). Such evidence would seem to account well for the repetition effects of the direct set. Reduced activity has been interpreted as reflecting decreased effort when the same targets repeat. This evidence is consistent with studies of neurological patients (Kristjánsson et al., 2005; O’Shea et al., 2018; Walsh et al., 2000) and TMS studies (Campana et al., 2005, 2007).

With regard to the indirect set, a number of findings are relevant. Firstly, a very interesting finding was reported by Becker et al. (2014), where they found distinct neural mechanisms for effects of feature change versus change in the relevant feature dimension. Relatedly, DiQuattro and Geng, (2011) found activity modulations in regions specifically associated with the changes of context, that then subsequently affect the activity of feature specific networks. Then there is evidence that changes in the attentional set are reflect activity modulations by nucleus accumbens (Woodward et al., 2006) and in the dorsolateral prefrontal cortex (Rudorf & Hare, 2014), an area often associated with the operation of visual working memory. We speculate that these mechanisms are responsible for the effects of repetition of the indirect set, and importantly, that they could be responsible for tuning the activity of neural networks that are more feature-specific.

We believe that the first-order effects in our paradigm will be accompanied by repetition suppression in feature-specific areas, while the indirect set will be generated by task set effects in areas such as anterior cingulate cortex and dorsolateral prefrontal cortex. These effects will then influence attentional and stimulus-specific areas that generate a top-down guidance signal guiding attention towards particular stimulus features. In any case, we believe that our new task is ideally suited to addressing questions of this sort.

## Conclusions

Our study provides novel insights into direct and indirect attentional sets during target identification, in particular how repetition priming affects performance. Performance was faster for direct than indirect sets and slowest when the sets were mixed within blocks. Repetition priming was strong, mainly when both the set and the target were repeated. Other repetition cases led to priming, but only for direct attentional sets, with little priming for the indirect set. According to the ideal-observer analysis, repetition priming patterns for direct sets are consistent with a first-order memory mechanism tracking objects and colors. Different mechanisms from those involved in search driven by direct sets are used for search with indirect sets. The findings highlight how our ability to locate important items, such as when searching for specific objects in a cluttered environment, can be significantly slowed when we rely on indirect cues rather than direct recognition. This research has practical implications for improving efficiency in tasks like navigation, security screening, or even daily activities like organizing and locating personal items, where attentional strategies and prior experience play a key role in optimizing search performance.

## Supplementary Information

Below is the link to the electronic supplementary material.

Supplementary file1 (DOCX 386 KB)

## Data Availability

Experimental results are available at GitHub (https://github.com/alexander-pastukhov/priming-attentional-sets-geistesblitz) and, additionally, at OSF (https://osf.io/jftg4/).

## Code availability

Ideal observer models, and analysis notebooks necessary to preprocess data, fit models, and reproduce all data analysis and figures are all available at GitHub (https://github.com/alexander-pastukhov/priming-attentional-sets-geistesblitz) and, additionally, at the Open Science Framework (https://osf.io/jftg4/). Please note that some parts of the analysis notebooks assume the presence of already sampled draws and leave-one-out (LOO) criteria files that were not included due to their size. The reader can either run sampling scripts included in the repository or contact the authors, who will be happy to provide them via an online large file transfer tool.
