# Supplementary material for: Two routes to a target: Visual priming for direct and indirect attentional sets
Source: Mem Cognit. 2025 Dec 15;54(5):1569–91. doi: 10.3758/s13421-025-01826-6 (PMC13407948; doi:10.3758/s13421-025-01826-6)
Supplement: Supplementary file 1 — Supplementary file1 (DOCX 386 KB) [file 13421_2025_1826_MOESM1_ESM.docx]

# Electronic Supplementary Material

## Model comparison


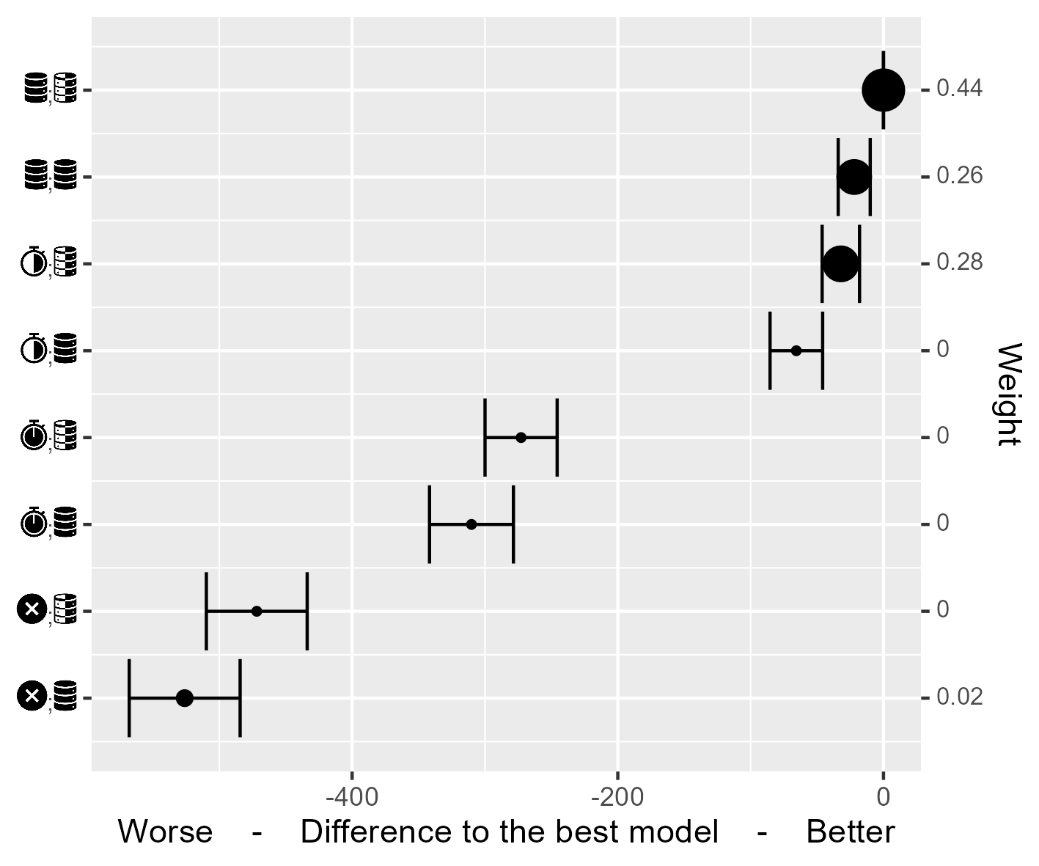


**Figure S1. *Easy*: Model comparison via leave-one out information criterion.** Difference and standard error of difference in ELPD relative to the best (top) model. Circle size and right y-axis show relative model weight computed via stacking algorithm (see Methods for details on LOO information criterion and stacking algorithm). Icons on the left depict model components (set and object only), please refer to **Table 3** for information.


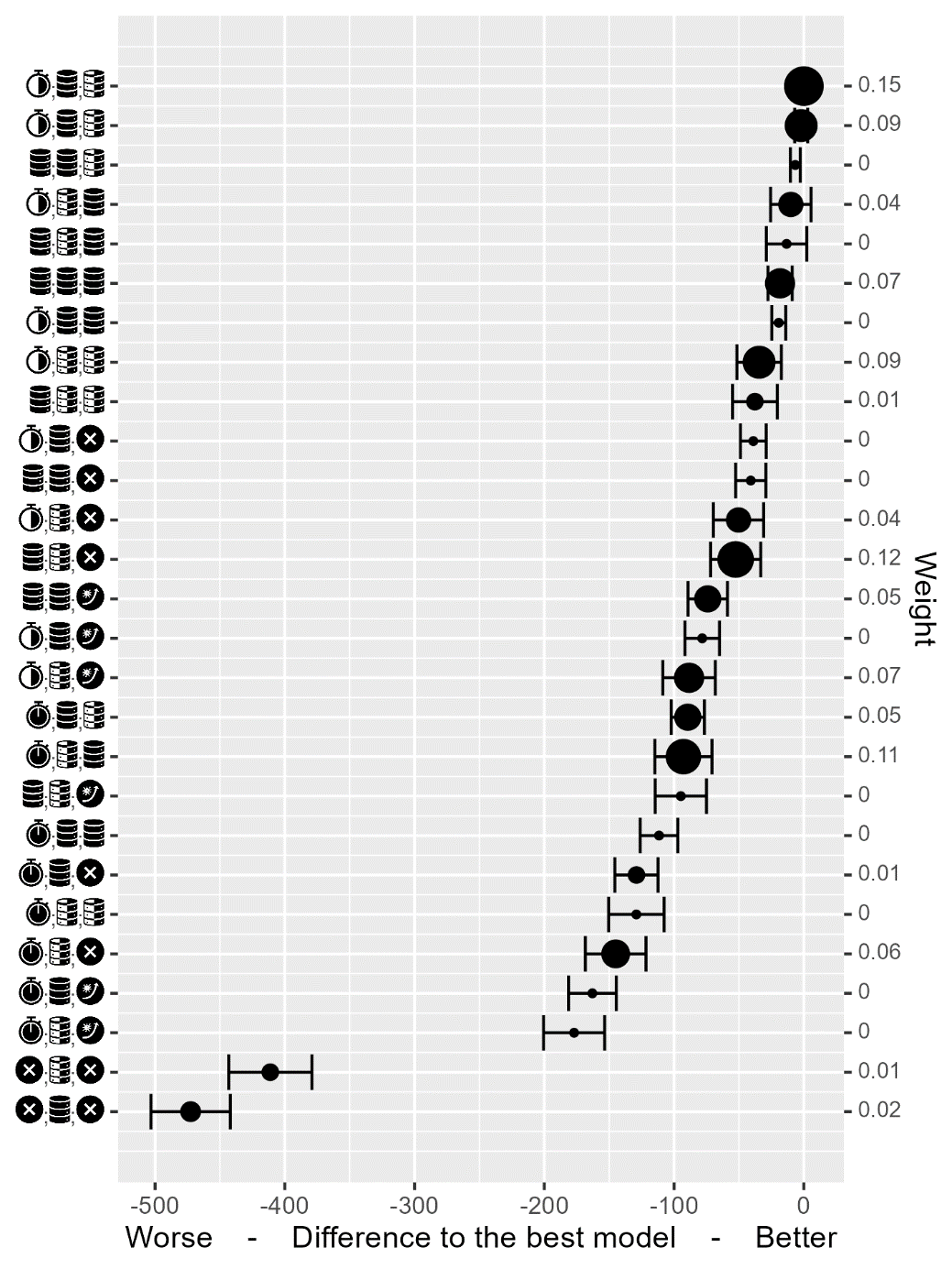


**Figure S2.** ***Medium*: Model comparison via leave-one out information criterion.** Difference and standard error of difference in ELPD relative to the best (top) model. Circle size and right y-axis show relative model weight computed via stacking algorithm (see Methods for details on LOO information criterion and stacking algorithm). Icons on the left depict model components (set, object, and color), please refer to **Table 3** for information.


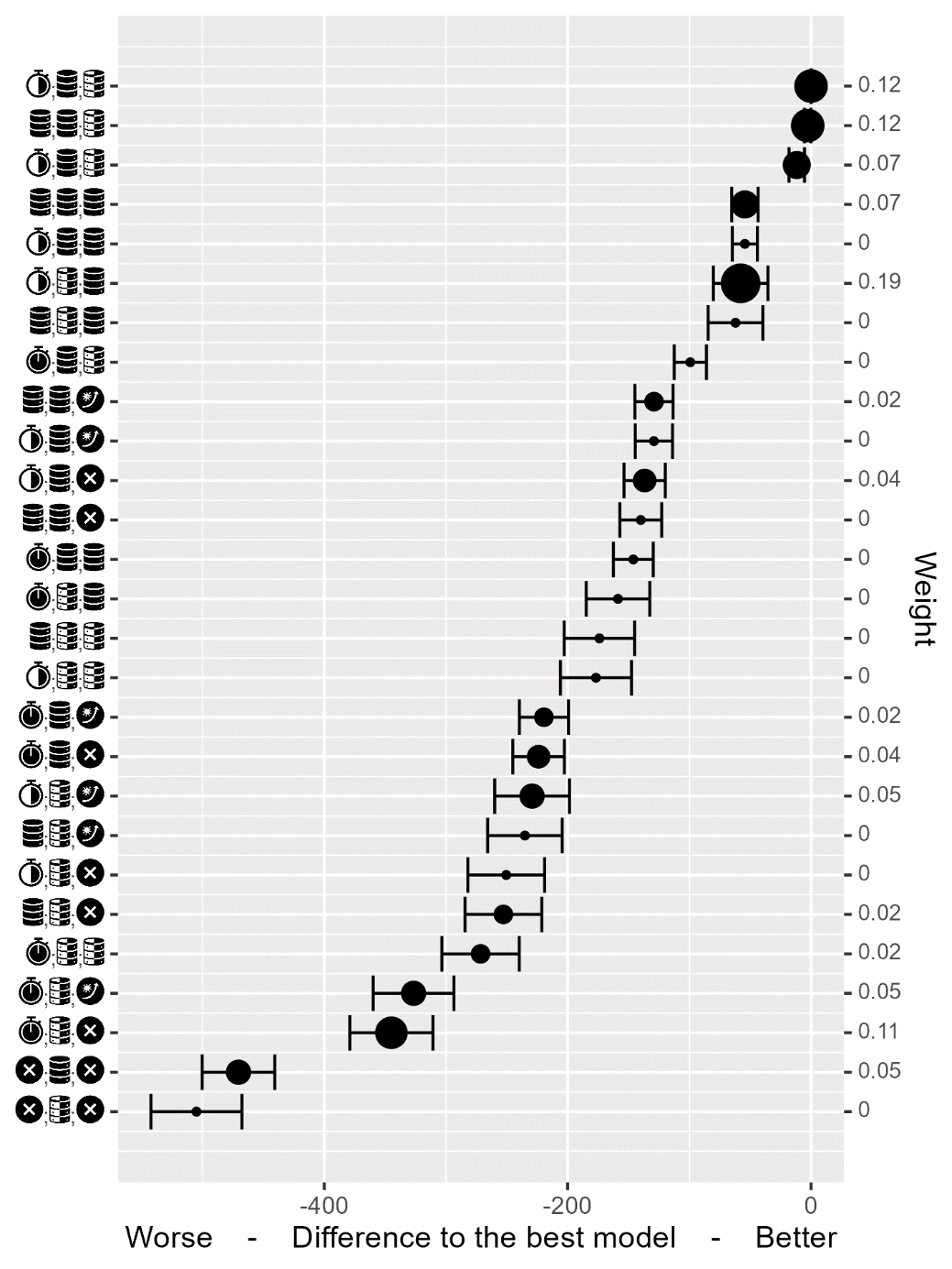


**Figure S3.** ***Hard*: Model comparison via leave-one out information criterion.** Difference and standard error of difference in ELPD relative to the best (top) model. Circle size and right y-axis show relative model weight computed via stacking algorithm (see Methods for details on LOO information criterion and stacking algorithm). Icons on the left depict model components (set, object, and color), please refer to **Table 3** for information.

## Analysis of subsets of trials, easy

**Table S1. *Easy*: repetition of attentional set and object.** Comparison of ideal observer models via leave-one-out information criterion. See **Table 3** and *Model* section in the main text for details on the correspondence between component labels and implemented mechanisms. ΔELPD refers to difference in the expected log predictive density relative to the best performing (top) model. Weight shows a relative model goodness-of-fit weight computed via stacking. Please refer to (Vehtari et al., 2017a) for details.

| **Set** | **Object** | **ΔELPD** | **Weight** |
| --- | --- | --- | --- |
| memory | common |  | 0.41 |
| memory | per set | -4.86±8.49 | 0.3 |
| different | common | -31.08±12.73 | 0.15 |
| different | per set | -38.88±15.61 | 0.12 |
| same | common | -243.91±25.11 | 0 |
| same | per set | -251.29±25.45 | 0 |
| none | common | -392.97±35.34 | 0.02 |
| none | per set | -412.29±35.52 | 0 |

**Table S2. *Easy*: repetition of object but not of an attentional set.** See caption of **Table S1** for details.

| **Set** | **Object** | **ΔELPD** | **Weight** |
| --- | --- | --- | --- |
| memory | per set |  | 0.33 |
| memory | common | -6.96±10.37 | 0.25 |
| different | per set | -12.9±8.65 | 0.25 |
| different | common | -23.03±13.81 | 0.17 |
| same | per set | -141.05±13.57 | 0 |
| same | common | -153.48±17.23 | 0 |
| none | per set | -219.53±17.03 | 0 |
| none | common | -242.54±19.86 | 0 |

**Table S3. *Easy*: repetition of an attentional set but not of an object.** See caption of **Table S1** for details.

| **Set** | **Object** | **ΔELPD** | **Weight** |
| --- | --- | --- | --- |
| memory | common |  | 0.05 |
| different | common | -8.91±8.68 | 0.52 |
| memory | per set | -9.01±7.39 | 0.42 |
| different | per set | -27.65±12.07 | 0 |
| same | common | -116.26±13.43 | 0 |
| same | per set | -137.52±15.92 | 0 |
| none | common | -189.48±15.74 | 0 |
| none | per set | -220.26±18.69 | 0 |

## Analysis of subsets of trials, medium

**Table S4. *Medium*: repetition of attentional set and object.** See caption of **Table S1** for details.

| **Set** | **Object** | **Color** | **ΔELPD** | **Weight** |
| --- | --- | --- | --- | --- |
| different | common | per set |  | 0.21 |
| memory | common | per set | -6.67±3.23 | 0 |
| different | common | common | -8.39±4.37 | 0 |
| different | per set | common | -21.41±12.53 | 0.03 |
| memory | common | common | -21.67±7.77 | 0.06 |
| Memory | per set | common | -22.82±12.34 | 0 |
| different | per set | per set | -24.62±11.52 | 0.02 |
| different | common | none | -25.14±9.02 | 0 |
| Memory | per set | per set | -26.03±11.49 | 0.03 |
| Memory | common | none | -38.97±10.05 | 0 |
| different | per set | none | -41.38±14.21 | 0.06 |
| Memory | per set | none | -41.54±13.94 | 0.12 |
| Same | common | per set | -50.22±11.36 | 0.07 |
| Same | common | common | -58.02±12.61 | 0.03 |
| different | common | object boost | -61.1±11.57 | 0 |
| Same | per set | common | -65.33±18.57 | 0.12 |
| different | per set | object boost | -66.43±15.49 | 0.08 |
| Same | per set | per set | -66.61±15.07 | 0 |
| Memory | per set | object boost | -69.92±14.78 | 0 |
| Memory | common | object boost | -71.15±12.62 | 0.04 |
| Same | common | none | -73.69±14.7 | 0.04 |
| Same | per set | none | -83.89±17.4 | 0 |
| Same | common | object boost | -104.85±15.79 | 0 |
| Same | per set | object boost | -105.29±17.85 | 0 |
| None | common | none | -308.33±27.68 | 0.09 |
| None | per set | none | -317.95±28.51 | 0 |

**Table S5. *Medium*: repetition of object but not of an attentional set.** See caption of **Table S1** for details.

| **Set** | **Object** | **Color** | **ΔELPD** | **Weight** |
| --- | --- | --- | --- | --- |
| different | per set | common |  | 0.14 |
| memory | per set | common | -3.32±1.62 | 0 |
| same | per set | common | -4.36±7.3 | 0.14 |
| different | common | per set | -11.89±11.59 | 0.17 |
| memory | common | per set | -15.52±11.73 | 0 |
| different | common | common | -20.84±12.78 | 0 |
| memory | common | common | -21.44±13.22 | 0.11 |
| different | common | none | -21.68±13 | 0.04 |
| memory | common | none | -25.04±13.38 | 0 |
| same | common | per set | -30.27±13.18 | 0 |
| memory | common | object boost | -31.37±12.12 | 0 |
| different | common | object boost | -31.39±11.58 | 0 |
| different | per set | none | -41.28±6.82 | 0.09 |
| same | common | common | -41.93±14.44 | 0 |
| same | common | none | -43.73±14.63 | 0.12 |
| memory | per set | none | -44.39±6.94 | 0.01 |
| different | per set | per set | -44.81±6.91 | 0.03 |
| memory | per set | per set | -46.9±6.99 | 0.02 |
| same | common | object boost | -50.34±13.31 | 0.03 |
| different | per set | object boost | -53.69±7.4 | 0 |
| memory | per set | object boost | -60.52±7.46 | 0 |
| same | per set | none | -61.67±9.46 | 0.09 |
| same | per set | per set | -64.43±9.27 | 0 |
| same | per set | object boost | -69.87±9.42 | 0 |
| none | per set | none | -148.11±11.74 | 0 |
| none | common | none | -182.53±18.78 | 0 |

**Table S6. *Medium*: repetition of an attentional set but not of an object.** See caption of **Table S1** for details.

| **Set** | **Object** | **Color** | **ΔELPD** | **Weight** |
| --- | --- | --- | --- | --- |
| same | per set | common |  | 0.13 |
| same | common | none | -11.67±9.87 | 0.15 |
| different | common | none | -13.67±11.04 | 0.06 |
| same | common | common | -15.59±9.22 | 0 |
| different | per set | common | -19.84±7.23 | 0.18 |
| different | common | common | -20.51±10.42 | 0 |
| same | common | per set | -22.04±9.83 | 0 |
| memory | per set | common | -23.15±7.27 | 0 |
| same | per set | none | -23.24±6.98 | 0.01 |
| different | common | per set | -23.77±10.75 | 0.12 |
| same | common | object boost | -29.97±9 | 0.02 |
| same | per set | per set | -30.17±7.08 | 0.07 |
| memory | common | per set | -30.39±11.1 | 0 |
| memory | common | none | -32.64±12.12 | 0.01 |
| same | per set | object boost | -36.44±7.36 | 0 |
| memory | common | common | -37.51±12.4 | 0.07 |
| different | per set | none | -38.33±10.26 | 0.11 |
| different | common | object boost | -38.84±10.87 | 0 |
| memory | per set | none | -41.66±10.17 | 0 |
| different | per set | per set | -46.06±10.43 | 0.01 |
| memory | per set | per set | -47.74±10.32 | 0 |
| different | per set | object boost | -54.34±10.92 | 0.02 |
| memory | common | object boost | -55.53±12.13 | 0 |
| memory | per set | object boost | -61.54±10.82 | 0 |
| none | common | none | -96.35±13.09 | 0.04 |
| none | per set | none | -126.01±12.42 | 0 |

**Table S7. *Medium*: repetition of color and attentional set but not of an object.** See caption of **Table S1** for details.

| **Set** | **Object** | **Color** | **ΔELPD** | **Weight** |
| --- | --- | --- | --- | --- |
| same | common | common |  | 0.04 |
| same | common | none | -1.74±4.07 | 0.14 |
| same | common | per set | -2.9±3.26 | 0.07 |
| same | per set | common | -5.81±10.16 | 0.15 |
| different | common | none | -12.55±9.21 | 0.02 |
| different | common | common | -13.2±8.24 | 0 |
| different | common | per set | -13.82±9.16 | 0.15 |
| different | per set | common | -15.51±12.5 | 0.1 |
| memory | common | per set | -15.97±9.06 | 0 |
| memory | per set | common | -16.89±12.34 | 0.01 |
| same | common | object boost | -22.69±5.26 | 0.01 |
| memory | common | common | -23.37±9.14 | 0.05 |
| memory | common | none | -23.64±9.52 | 0 |
| same | per set | per set | -28.54±10.01 | 0 |
| same | per set | none | -30.41±10.85 | 0 |
| different | common | object boost | -33.19±9.64 | 0 |
| different | per set | per set | -36.01±13.93 | 0 |
| memory | per set | per set | -36.21±13.79 | 0.06 |
| different | per set | none | -36.87±14.35 | 0.12 |
| memory | per set | none | -38.27±13.97 | 0 |
| memory | common | object boost | -43.32±10.22 | 0.01 |
| same | per set | object boost | -43.87±11.38 | 0 |
| different | per set | object boost | -50.46±14.93 | 0.03 |
| memory | per set | object boost | -53.96±14.24 | 0 |
| none | common | none | -147.76±16.16 | 0.05 |
| none | per set | none | -180.75±19.38 | 0 |

## Analysis of subsets of trials, hard

**Table S8. *Hard*: repetition of attentional set and object.** See caption of **Table S1** for details.

| **Set** | **Object** | **Color** | **ΔELPD** | **Weight** |
| --- | --- | --- | --- | --- |
| different | common | per set |  | 0.22 |
| memory | common | per set | -3.56±4.52 | 0.05 |
| different | common | common | -11.27±7.96 | 0 |
| memory | common | common | -16.47±10.73 | 0.09 |
| different | per set | common | -48.96±17.36 | 0.13 |
| memory | per set | common | -49.27±17.36 | 0 |
| same | common | per set | -56.89±10.55 | 0 |
| same | common | common | -65.89±13.17 | 0 |
| different | common | object boost | -73.8±12.81 | 0.01 |
| memory | common | object boost | -78.6±14.27 | 0.03 |
| different | per set | per set | -97.53±19.53 | 0.01 |
| memory | per set | per set | -97.77±19.5 | 0.13 |
| different | common | none | -97.9±14.62 | 0 |
| memory | common | none | -101.19±15.96 | 0 |
| same | per set | common | -102.05±20.01 | 0.09 |
| same | common | object boost | -128.99±16.46 | 0 |
| same | per set | per set | -143.61±20.91 | 0.01 |
| same | common | none | -144.84±17.88 | 0.06 |
| memory | per set | object boost | -146.64±21.22 | 0 |
| different | per set | object boost | -155.03±22.21 | 0 |
| different | per set | none | -173.69±22.8 | 0 |
| memory | per set | none | -175.52±22.72 | 0 |
| same | per set | object boost | -189.13±22.51 | 0.06 |
| same | per set | none | -214.57±23.97 | 0.04 |
| none | common | none | -315.03±26.42 | 0.07 |
| none | per set | none | -390.74±31.32 | 0 |

**Table S9. *Hard*: repetition of object but not of an attentional set.** See caption of **Table S1** for details.

| **Set** | **Object** | **Color** | **ΔELPD** | **Weight** |
| --- | --- | --- | --- | --- |
| different | common | per set |  | 0.14 |
| memory | common | per set | -1.09±3.52 | 0 |
| memory | common | common | -6.87±9.2 | 0.05 |
| memory | common | none | -7.01±8.37 | 0.04 |
| same | common | per set | -7.75±5.46 | 0 |
| different | common | none | -10.69±6.35 | 0.04 |
| different | common | common | -12.1±7.99 | 0.06 |
| memory | common | object boost | -12.37±10.84 | 0.07 |
| same | common | none | -16.08±7.94 | 0.07 |
| different | common | object boost | -17.18±10.03 | 0 |
| same | common | common | -18.65±9.07 | 0 |
| same | common | object boost | -23.33±10.88 | 0.05 |
| different | per set | common | -26.07±18.32 | 0.15 |
| memory | per set | common | -29.41±18.33 | 0 |
| same | per set | common | -32.02±18.9 | 0.03 |
| none | common | none | -141.23±12.04 | 0 |
| different | per set | none | -148.17±26.94 | 0.02 |
| memory | per set | none | -151.51±26.91 | 0 |
| same | per set | none | -156.34±27.34 | 0.13 |
| different | per set | per set | -160.55±27.34 | 0.1 |
| memory | per set | per set | -163.14±27.3 | 0 |
| memory | per set | object boost | -165.36±27.31 | 0 |
| different | per set | object boost | -167.61±27.71 | 0 |
| same | per set | per set | -169.87±27.77 | 0 |
| same | per set | object boost | -171.89±27.71 | 0.06 |
| none | per set | none | -210.68±26.64 | 0 |

**Table S10. *Hard*: repetition of an attentional set but not of an object.** See caption of **Table S1** for details.

| **Set** | **Object** | **Color** | **ΔELPD** | **Weight** |
| --- | --- | --- | --- | --- |
| different | common | common |  | 0 |
| same | common | common | -0.01±5.74 | 0 |
| same | common | per set | -8.05±8.53 | 0 |
| memory | common | common | -8.33±6.29 | 0.04 |
| different | common | per set | -8.37±6.4 | 0.12 |
| memory | common | per set | -12.74±7.24 | 0 |
| same | per set | common | -15.87±14.98 | 0.11 |
| same | common | object boost | -17.26±8.45 | 0.03 |
| different | common | object boost | -18.42±6.4 | 0 |
| same | common | none | -21.23±9.57 | 0.1 |
| different | common | none | -23.66±7.5 | 0.07 |
| memory | common | object boost | -24.83±8.45 | 0.08 |
| different | per set | common | -25.62±14.92 | 0.19 |
| memory | per set | common | -26.31±14.85 | 0.01 |
| memory | common | none | -31.83±9.34 | 0.04 |
| same | per set | per set | -86.22±20.51 | 0.04 |
| none | common | none | -92.34±12.15 | 0 |
| same | per set | none | -95.17±20.33 | 0.05 |
| same | per set | object boost | -97.24±21.02 | 0.05 |
| different | per set | per set | -101.63±21.3 | 0 |
| memory | per set | per set | -101.77±21.21 | 0.08 |
| different | per set | none | -112.27±21.03 | 0 |
| memory | per set | object boost | -112.95±21.54 | 0 |
| memory | per set | none | -113.21±20.89 | 0 |
| different | per set | object boost | -120.38±22.54 | 0 |
| none | per set | none | -194.29±23.88 | 0 |

**Table S11. *Hard*: repetition of color and attentional set but not of an object.** See caption of **Table S1** for details.

| **Set** | **Object** | **Color** | **ΔELPD** | **Weight** |
| --- | --- | --- | --- | --- |
| different | common | per set |  | 0.25 |
| memory | common | per set | -0.04±3.73 | 0.04 |
| same | common | per set | -2.83±7.87 | 0 |
| different | common | common | -7.99±5.75 | 0 |
| same | common | common | -11.25±9.9 | 0 |
| memory | common | common | -11.99±9.03 | 0.08 |
| different | per set | common | -31.74±13.37 | 0.07 |
| memory | per set | common | -32.24±13.37 | 0.01 |
| different | common | object boost | -33.92±7.94 | 0 |
| same | per set | common | -38.73±15.82 | 0.13 |
| memory | common | object boost | -38.84±10.27 | 0.04 |
| same | common | object boost | -40.13±11.45 | 0 |
| same | common | none | -56.72±13.05 | 0.09 |
| memory | common | none | -58.35±12.2 | 0.03 |
| different | common | none | -59.89±10.65 | 0 |
| same | per set | per set | -88.05±18.08 | 0.12 |
| memory | per set | per set | -88.91±17.16 | 0.03 |
| different | per set | per set | -89.05±17.25 | 0.03 |
| same | per set | object boost | -115.11±18.76 | 0 |
| memory | per set | object boost | -116.87±17.9 | 0 |
| different | per set | object boost | -123.37±18.77 | 0 |
| same | per set | none | -132.15±19.73 | 0.06 |
| different | per set | none | -135.8±19 | 0 |
| memory | per set | none | -135.98±18.87 | 0 |
| none | common | none | -171.32±18.49 | 0.02 |
| none | per set | None | -250.06±24.71 | 0 |
